# Hyperglycemia-induced oxidative stress and heart disease-cardioprotective effects of rooibos flavonoids and phenylpyruvic acid-2-*O*-β-D-glucoside

**DOI:** 10.1186/s12986-017-0200-8

**Published:** 2017-07-10

**Authors:** Phiwayinkosi V. Dludla, Elizabeth Joubert, Christo J.F. Muller, Johan Louw, Rabia Johnson

**Affiliations:** 1 Biomedical Research and Innovation Platform (BRIP), South African Medical Research Council, P.O. Box 19070, Tygerberg, 7505 South Africa; 2 Division of Medical Physiology, Faculty of Health Sciences, Stellenbosch University, Tygerberg, South Africa; 3 Plant Bioactives Group, Post-Harvest and Wine Technology Division, Agricultural Research Council (ARC) Infruitec- Nietvoorbij, Stellenbosch, South Africa; 4 Department of Food Science, Stellenbosch University, Stellenbosch, South Africa; 5 Department of Biochemistry and Microbiology, University of Zululand, KwaDlangezwa, South Africa

**Keywords:** Diabetes mellitus, Hyperglycemia, Oxidative stress, Cardiomyopathy, Rooibos, Polyphenols

## Abstract

Diabetic patients are at an increased risk of developing heart failure when compared to their non-diabetic counter parts. Accumulative evidence suggests chronic hyperglycemia to be central in the development of myocardial infarction in these patients. At present, there are limited therapies aimed at specifically protecting the diabetic heart at risk from hyperglycemia-induced injury. Oxidative stress, through over production of free radical species, has been hypothesized to alter mitochondrial function and abnormally augment the activity of the NADPH oxidase enzyme system resulting in accelerated myocardial injury within a diabetic state. This has led to a dramatic increase in the exploration of plant-derived materials known to possess antioxidative properties. Several edible plants contain various natural constituents, including polyphenols that may counteract oxidative-induced tissue damage through their modulatory effects of intracellular signaling pathways. Rooibos, an indigenous South African plant, well-known for its use as herbal tea, is increasingly studied for its metabolic benefits. Prospective studies linking diet rich in polyphenols from rooibos to reduced diabetes associated cardiovascular complications have not been extensively assessed. Aspalathin, a flavonoid, and phenylpyruvic acid-2-*O*-β-D-glucoside, a phenolic precursor, are some of the major compounds found in rooibos that can ameliorate hyperglycemia-induced cardiomyocyte damage in vitro. While the latter has demonstrated potential to protect against cell apoptosis, the proposed mechanism of action of aspalathin is linked to its capacity to enhance the expression of nuclear factor (erythroid-derived 2)-like 2 (*Nrf2*) expression, an intracellular antioxidant response element. Thus, here we review literature on the potential cardioprotective properties of flavonoids and a phenylpropenoic acid found in rooibos against diabetes-induced oxidative injury.

## Background

The prevalence of diabetes mellitus (DM) is increasing at an alarming rate worldwide. According to recent data by the International Diabetes Federation, the number of individuals living with DM is 415 million, and this figure is estimated to reach 642 million by the year 2040 [1]. Type 2 DM, which is associated with insulin resistance and obesity, represents approximately 90% of diabetic cases worldwide [2]. Type 1 DM is characterized by deficient insulin secretion and chronic hyperglycemia. Chronic hyperglycemia remains the leading causal factor for the development of cardiovascular disease (CVD) and heart failure (HF) in a diabetic state [3, 4]. Chronic hyperglycemia alters the myocardial substrate preference in cardiomyocytes and augments production of free radical species, giving rise to oxidative stress [5]. Oxidative stress may directly induce cardiac structural remodeling, a prominent sign of diabetic cardiomyopathy (DCM) [6, 7]. DCM is a distinct clinical entity that was first described about four decades ago [7]. The diagnosis of DCM remains nebulous and the precise mechanism explaining DCM has been partially explained. Although they remain mainly non-ischemic, distinctively affecting the heart muscle, cardiomyopathies play a pre-dominant role in inducing HF and are one of the major causes of death in Africa [8, 9]. Currently, there is no specific treatment for DCM; however, therapeutic drugs certainly play a significant role in the treatment of diabetes and its cardiovascular complications.

Insulin and metformin are the commonly used therapies for the treatment of DM and have been shown to present limited cardiac protective properties [10–12]. It is known that CVD-related deaths in individuals with DM are still a major concern [13]. Furthermore, lifestyle intervention of restricted energy intake and physical activity in persons with impaired glucose tolerance has been shown to improve CVD function. Nonetheless, most individuals do not adhere to such lifestyle interventions. On the other hand, antioxidants are among the leading therapies being investigated for their efficacy against various metabolic complications [14, 15]. In the last decade, there has been much interest in the potential health benefits of plant polyphenols, such as resveratrol, mangiferin and aspalathin as dietary antioxidants [16–18]. The rooibos flavonoid and dihydrochalcone, aspalathin, has been investigated and reported to contribute to the antidiabetic properties of rooibos extract as reviewed by Muller and colleagues [18]. This flavonoid and others have been shown to exert their therapeutic effects by mainly regulating the expression of key genes involved in energy metabolism and oxidative stress. Prime examples include 5′-adenosine monophosphate-activated protein kinase (AMPK), which is crucial for maintenance of myocardial substrate metabolism and nuclear factor (erythroid-derived 2)-like 2 (Nrf2), a transcription factor that is upregulated in response to oxidative stress [18, 19]. This review will discuss the cardioprotective potential of rooibos flavonoids and the phenylpropenoic acid, phenylpyruvic acid-2-*O*-β-D-glucoside (PPAG), against hyperglycemia-induced injury and heart disease. Physiological context is provided by a short overview of the role of oxidative stress in a diabetic heart.

### Mechanisms of oxidative stress leading to cardiac tissue damage

**Fig. 1:**
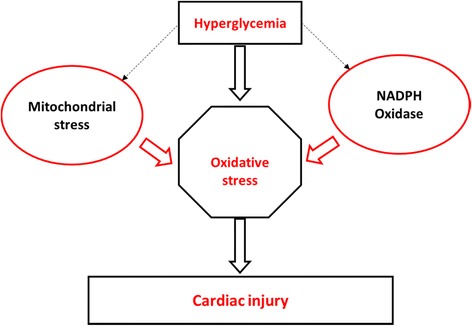
Chronic hyperglycemia is strongly associated with enhanced oxidative stress-induced myocardial injury. The mitochondrial electron transport chain and the actions of the nicotinamide adenine dinucleotide phosphate-oxidase (NADPH oxidase) enzymes remain the foremost sources of stress in cardiomyocytes

Chronic hyperglycemia is strongly associated with enhanced oxidative stress-induced myocardial injury [13, 20]. This has been confirmed by various laboratory studies showing strong correlation between oxidative stress and matrix remodeling in cardiomyocytes isolated from diabetic heart tissue [21, 22]. Oxidative stress is aggravated by enhanced levels of Reactive Oxygen Species (ROS) within cardiomyocytes [21, 23, 24]. Abnormal ROS production elicits an increased pro-inflammatory response resulting in myocardial apoptosis [25]. Some of the well-known reactive oxygen substances, associated with myocardial damage include superoxide anion (O_2_^•−^) and hydrogen peroxide (H_2_O_2_). Generation of ROS is generally a cascade of reactions that starts with the formation of O_2_^•−^ [26]. Superoxide anion rapidly dismutates to H_2_O_2_, either spontaneously or catalytically by superoxide dismutase (SOD), while H_2_O_2_ is decomposed by catalase (CAT) to water and oxygen. However, the mitochondrial electron transport chain and the actions of the nicotinamide adenine dinucleotide phosphate-oxidase (NADPH oxidase; Nox) enzymes remain the foremost sources of stress in cardiomyocytes (Fig. 1). Mitochondrial structural modification is affiliated with reduced endogenous antioxidant status in cardiomyocytes from diabetic heart tissue [24, 27]. Correspondingly, augmented activity of NADPH oxidase is demonstrated in the myocardium of diabetic animals at the same rate as oxidative damage [28].

The mitochondrion is an essential organelle for intracellular energy production. Increasing the cellular demand of the mitochondrion to produce energy is associated with accelerated ROS production. Given that a diabetic heart has a diminished mitochondrial antioxidant capacity [29], it is therefore not surprising that minor alterations in mitochondrial structure or function induced by increased ROS are associated with major changes in the heart muscle [30]. Increased ROS and mitochondrial depolarization, subsequent to diastolic dysfunction, have been reported in patients with metabolic disturbances [31, 32]. However, data explaining the precise role of mitochondrial dysfunction in a diabetic heart are still lacking.

Concurrent with energy generation is the constant generation of ROS within the mitochondria [33]. Accumulation of these radicals results in the induction of mitochondrial permeability transition (MPT) and the opening of high conductance permeability transition pores [34]. MPT formation has been reported to lead to an altered redox state of the mitochondria [30]. With disease states, the MPT opening is unique for being nonselective and allowing for the accumulation of excessive calcium (Ca^2+^) and other toxic compounds [35, 36]. The fate of the cell after an insult depends on the extent of MPT pore formation [36–38]. If MPT pore formation occurs only to a limited extent, the cell may recover through cell recovery mechanisms such as activation of mitophagy/ubiquitination, whereas if MPT pore formation is exacerbated, it accelerates apoptosis [38]. If it occurs to an even larger degree, the cell is likely to undergo necrotic cell death [38]. Thus, prevention of mitochondrial membrane depolarization may play a role in reducing myocardial injury associated with chronic hyperglycemia.

On the other hand, Nox is another system that plays a notable role in the generation of ROS in many cell types, including cardiomyocytes. Nox generates intracellular ROS by transferring electrons from NADPH across the cell membrane and coupling these to molecular oxygen to produce O_2_^•−^. Nox exists in different isoforms, i.e. Nox1 to Nox4, with Nox2 and Nox4 predominant in the heart muscle [39, 40]. Nox1 has been identified to be the major source of ROS production in vascular tissues, resulting in low levels of nitric oxide [41, 42]. Decreased levels of nitric oxide are connected to impaired endothelium-dependent vasodilation of coronary arteries [43]. Human aortic endothelial cells exposed to high glucose concentrations display amplified expression of Nox1 concomitant to enhanced oxidative damage [44]. The same study showed that diabetic mice lacking Nox1 had profound anti-atherosclerotic outcome related to reduced ROS generation. Although Nox2 and Nox4 are predominant in the heart muscles, these two ROS producing enzymes are also expressed in other cell types and are implicated in agonist-stimulated redox-sensitive signal transduction [39]. Nox2 has been shown to play a central role in insulin resistance-mediated oxidative damage in vascular tissue [45, 46]. Nox2 knockout transgenic mice with endothelial-specific insulin resistance present reduced ROS production and vascular dysfunction [45]. On the other hand, the Nox4 isoform is specifically expressed in mitochondria of cardiomyocytes; and mice lacking the Nox4 gene show reduced free radical damage [28]. Its overexpression in the mouse heart deteriorates cardiac dysfunction by initiating apoptosis through cytochrome c release [28]. Cytochrome c is a vital component of the mitochondrial electron transport chain, acting as an electron shuttle during redox generation of ATP [47]. Its release and mitochondrial depolarization are considered key physiological events of apoptosis [48]. In cardiomyocytes exposed to high glucose concentrations, cytochrome c release is enough to cause apoptosis, independent of mitochondrial depolarization status [49]. Complex systems within the apoptotic pathway exacerbate myocardial injury through cytochrome c release [50]. Therefore, interventions that could inhibit pro-apoptotic proteins and mitochondrial cytochrome c release could salvage myocardial injury.

### Endogenous cardioprotective mechanisms

#### Activation of AMPK

**Fig. 2:**
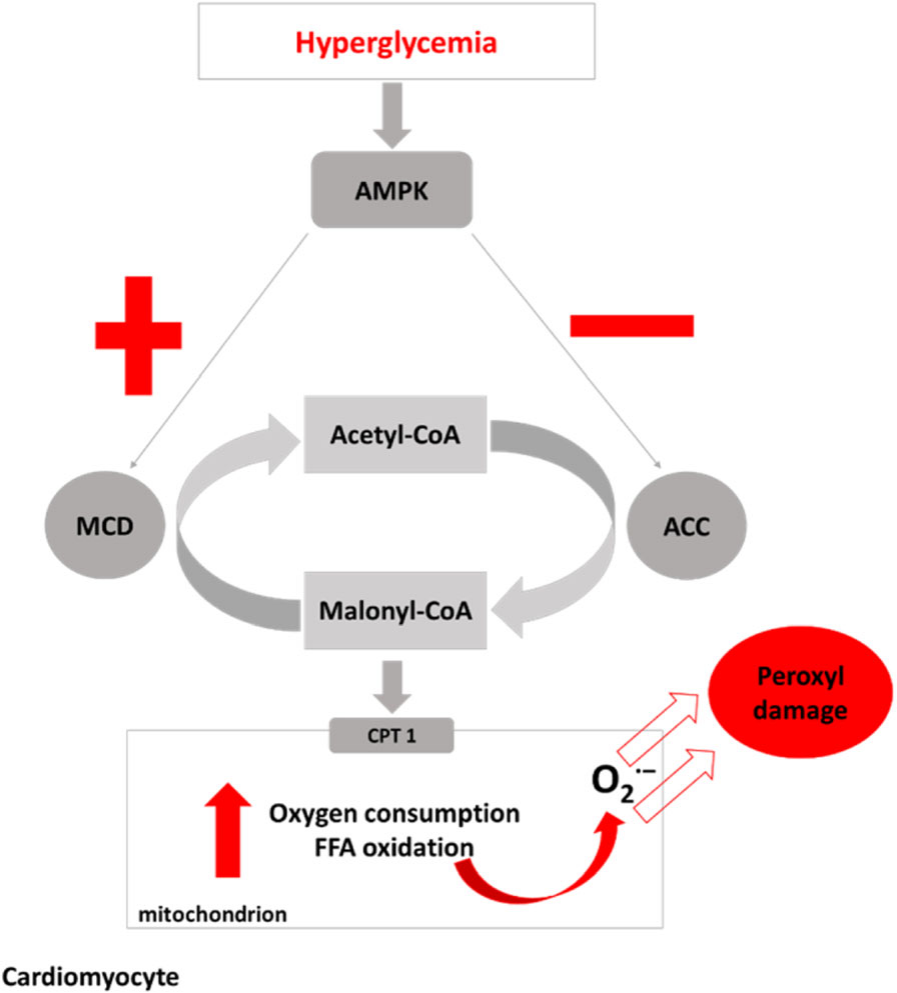
AMPK is associated with the phosphorylation of ACC and MCD to regulate myocardial energy metabolism. Chronic hyperglycemia activates AMPK, resulting in the phosphorylation of ACC, releasing the inhibitory effect of malonly-CoA on CPT1 and subsequently leading to enhanced entry of FFAs via CPT1 into the mitochondria for beta-oxidation. Abnormally increased beta-oxidation is further accountable for mitochondrial membrane damage through peroxyl radicals. Keys: ACC- acetyl-CoA carboxylase; AMPK- 5’ adenosine monophosphate (AMP)-activated protein kinase; CPT1- carnitine palmitoyltransferase 1; FFA- free fatty acid; MCD- malonyl-CoA decarboxylase

AMPK is a heterotrimeric protein composed of a catalytic alpha, non-catalytic beta and gamma subunit. The main function of this kinase is to preserve ATP or to promote alternative pathways of ATP generation. It functions as a sensor during low energy states such as ischemia to change substrate utilization and thereby increase or decrease ATP synthesis. Its activation is controlled by an increase in the AMP:ATP ratio [51]. Stimulation of AMPK leads to phosphorylation of many target proteins important for ATP synthesis and utilization while concurrently inhibiting ATP-consuming pathways such as fatty acid synthesis. In the diabetic heart, AMPK activation is linked to phosphorylation of both acetyl-CoA carboxylase and malonyl-CoA decarboxylase; however, its association with the latter in the heart remains to be fully elucidated [52–54]. Acetyl-CoA carboxylase and malonyl-CoA decarboxylase are both important for the interconversion of acetyl-CoA to malonyl-CoA (Fig. 2). Phosphorylation of acetyl-CoA carboxylase by AMPK reduces the generation of malonyl-CoA; thus promoting the entry of FFAs for beta-oxidation into mitochondria through carnitine palmitoyltransferase I [55]. Resultant increased levels of ATP and citrate through beta-oxidation are responsible for the allosteric inhibition of glycolysis through phosphofructokinase-1 [56]. This causes accelerated ROS production and associated membrane peroxidation [24, 26]. Hence, adequate control of uptake and oxidation of FFAs remain crucial for optimal functioning of the myocardium, especially in a diabetic state.

#### Activation of Nrf2

Free radicals within mitochondria are generally removed by mitochondrial SOD, thereby generating H_2_O_2_. This process allows H_2_O_2_ to be further reduced to water by glutathione (GSH) or CAT. GSH remains an important intracellular antioxidant to prevent free radical damage. GSH can easily be oxidized to its disulfide form during oxidation reactions; thus, NADP transhydrogenase enzymes remain important to maintain the reduced form of this co-factor [57]. NADP transhydrogenase functions by transferring electrons from a reduced form of nicotinamide adenine dinucleotide (NADH) to NADP^+^ in order to regenerate GSH [58]. Reduced expression of GSH is consistently reported in experimental models investigating a diabetic heart [21, 24]. This was confirmed when investigated in either human subjects at risk of CVD or mice that are chronically subjected to hyperglycemia and hyperlipidemia [59, 60].

**Fig. 3:**
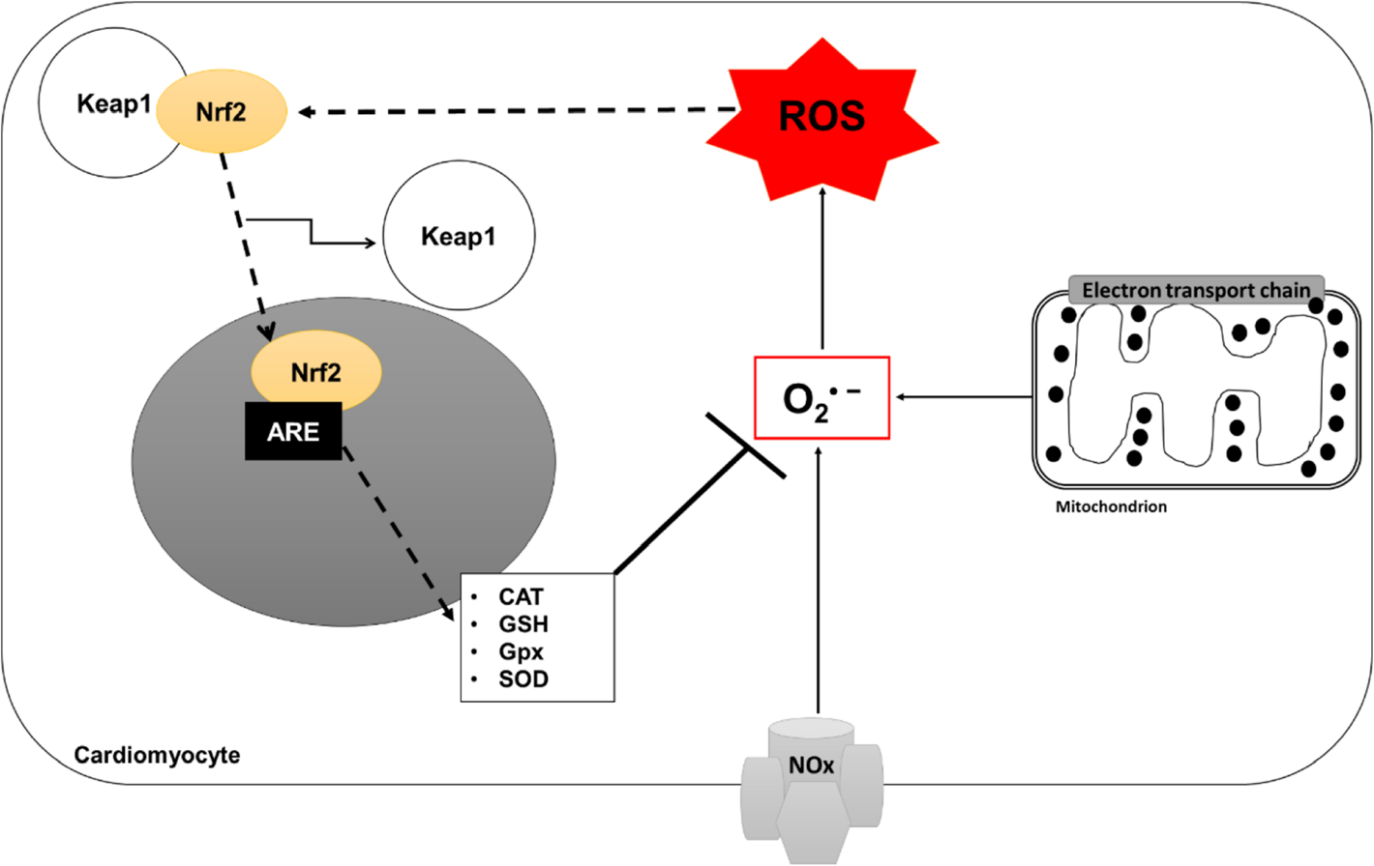
The role of Nrf2 in response to increased ROS within a diabetic heart. Nox and mitochondrial-ETC cause augmented production of O_2_^∙−^, which damages the cell through ROS. The cell reacts by activating the Nrf2-mediated antioxidant response system. Activated Nrf2 causes it to dissociate from Keap1 and migrate into the nucleus where it binds ARE and cause increased expression of cytoprotective genes and phase II detoxifying enzymes to eliminate ROS. Keys: ARE-antioxidant response element; CAT- catalase; Gpx- glutathione peroxidase; GSH- glutathione; Keap1- Kelch-like ECH-associated protein 1; Nox- NADPH oxidase; O_2_^∙−^ superoxide ion; Nrf2- nuclear factor (erythroid-derived 2)-like 2; ROS-reactive oxygen species

Expression of antioxidant response genes, including GSH is regulated by the redox-sensitive transcription factor, Nrf2 [61]. Nrf2 is a transcriptional regulator that is activated in response to intracellular stress (Fig. 3). Genes activated by Nrf2 can be classified into different groups, including phase II detoxifying and cytoprotective enzymes. Nrf2 resides in the cytoplasm, where it is subjected to continuous degradation by the ubiquitin-proteasome [62]. Under stressful conditions such as ischemia or oxidative stress, Nrf2 is activated by disassociating from its negative regulator Kelch-like ECH-associated protein 1 (Keap1) and translocates to the nucleus. Once in the nucleus, it forms a heterodimer with Maf protein before binding to the antioxidant response element (ARE) to initiate and activate antioxidant defence genes [63]. An overview of the pathway associated with the activation of Nrf2 and its protective effect against ROS in a cardiac cell is illustrated by Fig. 3. Activation of Nrf2 in epithelial cells has been shown to induce GSH synthesis and thus protects against oxidative stress [64]. In addition to its negative regulation of Nrf2, Keap1 also acts as a sensor for a wide array of stressors that could activate Nrf2. Significant down-regulation of cardiac Nrf2 expression is concomitant to increased ROS and reactive nitrogen species damage in hearts of diabetic animals [62–64]. Thus, agents that can significantly up-regulate Nrf2 expression have a potential to protect cardiomyocytes against high glucose-induced apoptosis.

#### Cardioprotective potential of current antidiabetic agents

Primary interventions that may salvage a diabetic heart at risk from myocardial infarction mainly target maintaining low blood sugar levels [13]. While such interventions are achievable and beneficial to the heart, adherence to lifestyle changes remains a big challenge. Therefore, antidiabetic agents that could suppress postprandial and chronic hyperglycemia may be effective in decreasing the risk of HF. Despite evidence on the efficacy of antidiabetic and antidyslipidemic drugs such as dipeptidyl peptidase-4 inhibitors and statins [65, 66], metformin remains the leading first line antidiabetic drug for type 2 diabetic individuals with known cardiac complications [67, 68]. In addition to its accomplished antidiabetic properties [69, 70], metformin is associated with improved clinical outcomes in diabetic patients with HF [71, 72]. Although clinical data are lacking, metformin enhances the efficacy of a number of synthetic drugs and novel medicinal compounds currently screened for metabolic benefits in vitro [73–75]. Metformin monotherapy or its use as an add-on effect to glibenclamide improves the intracellular antioxidant status of the myocardium in streptozotocin-induced diabetic Sprague-Dawley rats [76]. In addition to improving the antioxidant capacity of heart cells, metformin may benefit the heart by enhancing autophagy and inhibiting MPT opening [77, 78]. However, an increasing toll of cardiovascular related deaths in diabetic patients on treatment warrants further investigation into alternative treatment regimes.

#### Cardioprotective potential of rooibos

**Fig. 4:**
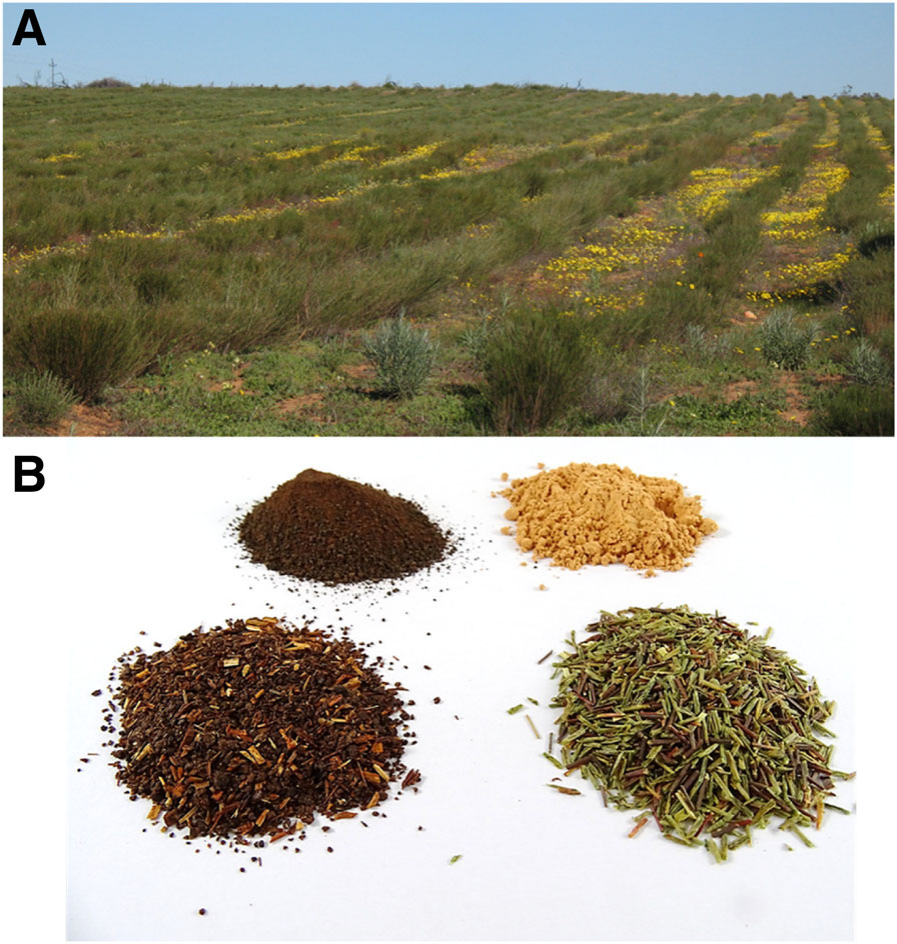
Photos of a rooibos plantation (**a**) and the two forms of processed plant material (**b**), fermented and unfermented rooibos with spray-dried powders of their hot water extracts. The fermentation process gives fermented rooibos its distinctive reddish-brown colour, while unfermented rooibos tea maintains its green colour

In recent years, the use of plant-derived products as a cardioprotective therapy is receiving increasing attention [59, 79, 80]. Rooibos (*Aspalathus linearis*) is an indigenous South African plant well-known for its potential health benefits. Rooibos tea is available in two forms: a “fermented” or oxidized form; and an “unfermented” or unoxidized form (Fig. 4). The unfermented product is also referred to as green rooibos. The “fermentation” process gives fermented rooibos its distinctive reddish-brown colour, while unfermented rooibos tea maintains its green colour (Fig. 4b). The fermentation process is important to develop the characteristic taste and aroma of rooibos tea, traditionally consumed [81]. Its effect on the health outcomes of rooibos is obscured if the products do not originate from the same bush due to large inherent variation in the phenolic content of the rooibos plant [81]. However, it is well established fermentation decreased the flavonoid content of rooibos [81]. Infusions and extracts prepared from unfermented rooibos have higher antioxidant capacity than those from fermented rooibos, largely due to higher levels of flavonoids, in particular aspalathin in the unoxidized plant material [81]. Consumption of a “ready-to-drink” unfermented rooibos beverage as opposed to one produced from fermented rooibos effected a 28% higher total radical-trapping antioxidant potential in the plasma of human subjects [82]. Nonetheless, both forms of rooibos are increasingly studied in experimental diabetes and related complications, given that fermented rooibos is more readily available and forms the bulk of rooibos production [81].

In a recent study, Oh and colleagues have demonstrated that the total flavonoid content of a water extract of rooibos, determined using a colorimetric assay based on aluminium complexation, is higher than that of lemongrass tea, mulberry leaf tea, bamboo leaf tea, lotus leaf tea, and persimmon leaf tea [83]. However, they further showed that the total flavonoid content of this extract is slightly lower than that of green and black tea. Von Gadow and colleagues have also previously shown that both fermented and unfermented rooibos, when tested together with green, oolong and black tea, present strong antioxidant properties in vitro [84]. They further showed that this antioxidant effect, as evaluated using the 2,2-diphenyl-1-picrylhydrazyl radical assay, was reduced in the order: green tea > unfermented rooibos > fermented rooibos > semifermented rooibos > black tea > oolong tea. Accompanying its well-documented antioxidant capacity [18, 84–89], rooibos has been shown to inhibit adipogenesis in vitro [90], reverse palmitate-induced insulin resistance in 3T3-L1 adipocytes [91] and prevent inflammation in vivo [92–94]. An aspalathin-enriched green rooibos extract, containing 18.4% aspalathin has demonstrated an even higher hypoglycemic potential through its inhibitory effect of alpha-glucosidase and suppressing fasting plasma glucose levels in animal models [91, 95]. Another green rooibos extract, containing 6.6% aspalathin promoted glucose transporter 4 translocation to the plasma membrane and suppressed advanced glycation end products (AGEs)-induced oxidative damage in cultured skeletal L6 muscle cells, pancreatic beta-cells and obese diabetic KK-Ay mice [96]. Rooibos inhibits experimentally-induced oxidative stress [21]; and improved cardiovascular function by reducing lipid peroxidation, blood pressure and angiotensin-converting (ACE) enzyme in various experimental models [97–100]. Moreover, it protected against ischemia-reperfusion injury by modulating the phosphatidylinositol 3-kinase/protein kinase B (PI3K-AKT) pathway [101]. Controlling free fatty acid oxidation by modulating phosphorylation of AMPK remains central in the preventive effect of rooibos against diabetes associated cardiac complications [90, 91, 96]. The cardioprotective potential of rooibos is summarized in Table 1.

**Table 1:** The cardioprotective effect of rooibos, its flavonoids and a phenylpropenoic acid

Rooibos/compounds	Model	Experimental outcome	References
Rooibos	Aqueous extract of fermented rooibos on cardiomyocytes isolated from diabetic rats	Prevented experimentally induced oxidative stress and ischemia	[21]
	Fermented rooibos tea for 6 weeks in human subjects at risk of cardiovascular disease (CVD)	Reduced CVD risk by improving lipid profile and redox status	[59]
	Aqueous extract of fermented rooibos in endothelial cells from human umbilical veins (HUVECs)	Prevented vascular-induced inflammation by enhancing nitric oxide production	[86, 92, 93]
	Aqueous extract of fermented rooibos on non-diabetic rats	Acted as a bronchodilator, antispasmodic and blood pressure lowering effects	[97]
	Fermented rooibos tea in healthy human subjects	Prevented myocardial infarction by inhibiting angiotensin-converting enzyme (ACE)	[98–100]
	Aqueous extracts of fermented and unfermented rooibos in non-diabetic rats	Reversed ischemia-reperfusion injury	[101]
Aspalathin and nothofagin	Aspalathin and nothofagin on high glucose-induced vascular in HUVECs and mice	Prevented inflammation and thrombosis by suppressing TNF-α, IL-6 and NF-κB	[118, 119]
	Aspalathin in H9c2 cardiomyocytes exposed to high glucose and cardiomyocytes isolated from insulin resistant rats	Prevented cell apoptosis by reducing phosphorylation of AMPK; decreasing inflammation and lipid accumulation; and attenuated oxidative damage via increasing *Nrf2* expression	[73, 124–126]
Orientin and isoorientin	Orientin on isolated hearts of nondiabetic rats, rabbits and guinea pigs as well as H9c2 cells	Prevented ischemia-reperfusion injury and platelet aggregation by inhibiting mPTP formation and apoptosis	[137–139]
Orientin and isoorientin	Orientin on rats	Prevented myocardial infarction	[134]
	Isoorientin in low density lipoprotein isolated from human plasma	Prevented formation of atherosclerotic lesions by inhibiting low density lipoprotein (LDL) oxidation	[135]
	Orientin in non-diabetic rats	Attenuated ventricular remodeling associated with myocardial infarction	[136]
	Orientin and isoorientin in lipopolysaccharide-induced reperfusion injury	Protected vascular barrier integrity by inhibiting hyperpermeability	[223]
Vitexin and isovitexin	Vitexin on primary cardiomyocytes and isolated rat hearts and on rats	Prevented ischemia-reperfusion injury by reducing calcium overload and modulating ERK1/2 signaling and MAPK pathway	[146, 149, 224]
	Vitexin on primary rat cardiomyocytes	Prevented cardiac hypertrophy by inhibiting calcineurin and CaMKII signaling pathways	[151]
	Vitexin on dogs	Reduced aortic pressure, arterial and pulmonary capillary pressure and heart rate	[150, 225]
	Vitexin on rats	Attenuated acute doxorubicin cardiotoxicity by reducing oxidative stress and apoptosis	[226]
Luteolin and chrysoeriol	Luteolin on isolated rat cardiomyocytes, rabbit hearts and anesthetized pigs	Prevented ischemia-reperfusion injury and enhanced relative coronary flow	[157, 159, 162]
	Luteolin on rat endothelium-denuded aortic rings	Induced vasorelaxion by regulating calcium and potassium channels and reducing oxidative stress	[227]
	Luteolin on vascular smooth muscle cells and rats	Prevented hypertensive vascular remodeling	[160]
	Luteolin on diabetic and normal rats	Alleviated vascular complications associated with insulin resistance through the Pparγ pathway	[161]
Luteolin and chrysoeriol	Luteolin-7-glucoside on isolated primary rat cardiomyocytes	Prevented ischemia-reperfusion injury and increased of coronary flow	[228]
	Chrysoeriol in rats under anesthesia and H9c2 cells	Reduced arterial blood pressure and protected against doxorubicin-induced cardiotoxicity	[97, 172]
Quercetin and rutin	Quercetin on rats	Protected against diabetic cardiomyopathy, autoimmune myocarditis, LDL-oxidation, and doxorubicin-induced lipid peroxidation	[185–192]
	Quercetin in either endothelial cells or rats	Presented antihypertensive potential and reduced cardiac hypertrophy by increasing antioxidant capacity	[229–233]
Hyperoside and rutin	Hyperoside in vitro and in vivo	Protected against hyperglycemia induced inflammation	[208]
	Hyperoside in ECV304 cells	Prevented advanced glycation end products and promoted via the c-Jun N-terminal kinases (JNK) pathway	[205]
	Hyperoxide in vitro and in vivo	Hydrogen peroxide induced cell damage and ischemia reperfusion injury	[209, 211, 212]
	Rutin on rats	Protected against advanced glycation end products, oxidative stress and myocardial infarction	[199, 234, 235]
Phenylpyruvic acid-2-*O*-β-D-glucoside (PPAG)	PPAG on high-glucose exposed H9c2 cells	Protected against substrate impairment, mitochondrial depolarization and cell apoptosis	[221]

A profound relationship between a diet rich in polyphenols and health has given hope to long-term effective interventions that could prolong the onset of diabetes and its co-morbidities [102]. Flavonoids constitute a major sub-class of polyphenols, which can be further divided into different sub-groups such as dihydrochalcones, flavonols and flavones, the predominant rooibos flavonoid sub-groups. Variations in the hydroxylation pattern, glycosylation and chromane ring (Ring C) underpin their structural differences (Table 2). Structural features of flavonoids relevant for their antioxidant properties [103] also explain binding affinity to plasma proteins [104] and enzymes such as alpha-glucosidase [105]. Whilst in vitro data suggest flavonoid aglycones to be more effective than their glycosides, lack of in vivo data precludes broad generalizations concerning the effect of glycosylation on the benefits of flavonoids for human health [106].

**Table 2:**
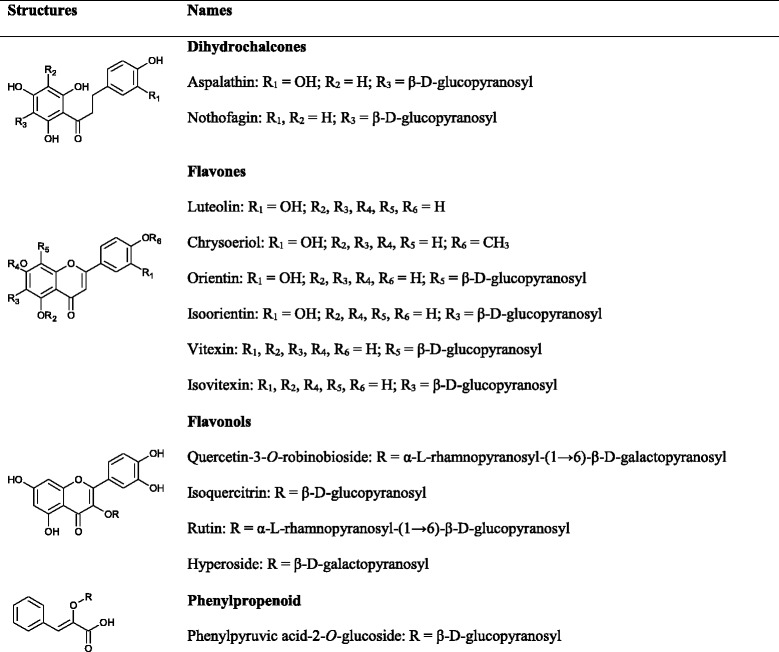
Molecular structures of flavonoids and a phenylpropenoid present in rooibos

In the following sections the focus will fall on flavonoids found in rooibos that have an ameliorative effect against various metabolic diseases. Of interest is the predominance of *C*-glucosyl flavonoids in rooibos, and in particular the dihydrochalcones (aspalathin and nothofagin) and their flavone derivatives (orientin, isoorientin, vitexin and isovitexin). The major flavonols are *O*-glycosyl derivatives of quercetin, i.e. quercetin-3-*O*-robinobioside, hyperoside, isoquercitrin, and rutin. The aglycones, luteolin, chrysoeriol and quercetin, are present in low to trace levels in rooibos [107]. Discussion of their bioactivities is included to underscore the health potential of rooibos.

PPAG is per definition not a phenolic compound due to the absence of a hydroxyl group on the phenyl ring. This compound is a biosynthetic precursor of flavonoids [108] and rooibos is one of the few plants demonstrated to date to be a substantial source.

#### Rooibos dihydrochalcones

The dihydrochalcone, aspalathin is unique to rooibos, while its 3-deoxy analogue, nothofagin, is relatively rare with its presence also confirmed in *Notofagus fusca* and *Schoepfia chinensis* [81]. Unfermented rooibos tea beverage contains 10-fold or more aspalathin and nothofagin when compared to the fermented product [107, 109]. This is not surprising as the fermentation process is known to reduce their content in rooibos [81, 110]. Despite *C*-glycosides having very low bioavailability due to the inability of intestinal enzymes to hydrolyze the C-C bond linking the sugar moiety to the aglycone and thus influencing their absorption process [111], aspalathin has been reported in the plasma of subjects who consumed 500 mL of green rooibos infusion, containing 287 mg aspalathin [112]. However, generally as reported for other dihydrochalcones [113, 114], the human gut microbiota can possibly enhance the absorption of aspalathin and nothofagin in the small intestine by splitting off the aglycone from the glucose moiety [115]. In vivo, the low levels of aspalathin are difficult to detect in serum, however metabolites (glucuronides and sulfates) of aspalathin and nothofagin have been detected in urine of human subjects 5 h after consumption of 500 mL of either fermented or unfermented rooibos [109]. Recent data have demonstrated that aspalathin is absorbed and metabolized in mice to mostly sulphate conjugates detected in urine, while the mode of absorption is hypothesized to occur through the monolayer paracellularly [116].

The biological of activity of aspalathin and nothofagin has been primary associated with their known strong antioxidant properties [73, 117]. Both compounds protected against high glucose-induced vascular inflammation and platelet aggregation when tested in endothelial cells and mice; however, nothofagin did not have any anticoagulant effect in mice [118, 119]. Increasing research is presented focusing on aspalathin and its enhanced efficacy to prevent metabolic-associated complications in vitro and in vivo models [18, 120–122]. Our laboratory has presented recent evidence that aspalathin reversed palmitate-induced insulin resistance in cultured adipocytes [123], while it prevented high glucose-inducedapoptosis by improving substrate metabolism in H9c2 cells exposed to high glucose or cardiomyocytes from insulin resistant rats [73, 124, 125]. In addition to regulating AMPK and enhancing *Nrf2* expression, aspalathin can modulate the expression of peroxisome proliferator-activated receptor gamma and sterol regulatory element-binding protein 1/2, transcriptional factors involved in lipid metabolism, in addition to inhibiting inflammation via interleukin-6/Janus kinase 2 pathways, leading to reduced myocardial apoptosis [73, 124, 126].

#### Rooibos flavones

The major flavones present in rooibos include orientin and isoorientin, the flavone derivatives of aspalathin, and vitexin and isovitexin, the flavone derivatives of nothofagin. Minor flavones include the aglycones, luteolin, and chrysoeriol (Table 2). Lower levels of flavones are present in fermented rooibos [81]. Food processing may also change their content. The orientin and isoorientin content of a ready-to-drink rooibos beverage showed a slight change as a result of pasteurization and storage, postulated to be due to the conversion of aspalathin to these compounds [110, 127]. Except for luteolin, there is very limited data on the characteristic metabolism and transportation of these flavone glucosides. The absorption of orientin, isoorientin and vitexin has been reported on Caco-2 cell monolayers [128, 129], with transporter mediated efflux in addition to passive diffusion shown to be the predominant mode of transportation. In a pharmacokinetics study using Sprague–Dawley rats, intravenous administration of a 20 mg/kg dose of orientin was found to be highly recovered in plasma and eliminated within 90 min after intravenous administration [130]. On the other hand, permeability and absorption rate of luteolin has been shown to be significantly greater in the colon and ileum compared to the duodenum and jejunum in rats [131]. Furthermore, some of these compounds, including isoorientin may be deglycosylated to their aglycones by gut microbiota as reviewed by Muller et al. [18].

The strong antioxidant properties of flavones have been associated with their free radical scavenging properties [132]. Although very few studies are available on the antidiabetic properties of orientin and isoorientin, extracts with abundant levels of vitexin, orientin and isoorientin have been shown to inhibit adipogenesis in 3T3-L1 adipocytes [90, 133]. Relevant to the cardiovascular system, these compounds have been reported to inhibit high glucose-induced vascular inflammation [134], atherosclerosis [135], cardiac remodeling [136] and ischemia-reperfusion injury [137–139]. Additional protective effects of these compounds are summarized in Table 1 and are mainly mediated by nuclear factor kappa B, a transcriptional factor involved in diabetic-induced HF [140]. Moreover, isoorientin has been shown to reduce other diabetic associated complications such as lipid toxicity and insulin resistance [141]. Together with direct radical scavenging activity, the protective mechanism of isoorientin has been linked to the induction of Nrf2 pathway-driven antioxidant response through phosphatidylinositol 3-kinase signaling [142]. On the other hand, oral administration of vitexin and isovitexin at 1 mg/kg has been shown to reduce postprandial blood glucose levels in sucrose loaded normoglycemic mice [143]. In addition to inhibiting α-glucosidase [144], vitexin and isovitexin rich extracts have been demonstrated to attenuate diabetes linked complications such as adipogenesis and AGEs in vitro [132, 145]. Vitexin reverses ischemia-reperfusion injury in perfused rat hearts and brain by attenuating inflammatory response and apoptosis [146–149]; it increased coronary artery blood flow and cardiac output in anesthetized animals [150]; and it improved cardiac hypertrophy by reducing the expression of calcium downstream effectors, calcineurin-NFATc3 and phosphorylated calmodulin kinase II (CaMKII), both in vitro and in vivo [151]. Other associated cardioprotective mechanisms of vitexin may include inhibiting cardiomyocyte apoptosis by reducing calcium overload and extracellular signal regulated kinase (ERK1/2) [152].

Furthermore, although available in trace levels in rooibos, release of luteolin from orientin and isoorientin in the gut may enhance the levels to physiological relevance. Accumulative evidence suggests strong ameliorative effect of luteolin against diabetes and CVD associated complications [153–155]. The antidiabetic properties of luteolin include improving hepatic insulin sensitivity by suppressing gluconeogenesis in diet-induced obese mice [153]; it prevented neuronal injury and cognitive performance by attenuating oxidative stress in rats [154]; and attenuated morphological destruction of the kidney in rats [155]. The anti-inflammatory properties of luteolin include inhibiting elevated levels of interleukin-1β and nuclear factor kappa B [156–158]. Relevant to the heart, luteolin reduced systolic and diastolic blood pressure of various animal models [159, 160]; it improved contractile function [161]; and blocked apoptosis following ischemia-reperfusion in adult rat cardiomyocytes via downregulating microRNA-208b-3p [162]; it attenuated HF in a rat model of DCM [163]; and protected against acute and chronic periods of isoproterenol-induced myocardial infarction by suppressing mitochondrial lipid peroxidation [164]. One of the important mechanisms linked to the cardioprotective effect of luteolin during ischemia-reperfusion injury include regulation of ERK1/2 and c-Jun N-terminal kinase (JNK), which are pathways implicated in generation of inflammation [165]. The number of functional hydroxyl groups on the structure of luteolin directly correlates to its scavenging effect of hydroxyl radicals [166].

Another flavone of interest that is present in very low quantities in rooibos is chrysoeriol [167]. Chrysoeriol has been previously shown to be more effective in the protection against lipid peroxidation than its glycoside (chrysoeriol-6-*O*-acetyl-4′-β-D-glucoside) when tested in vitro [168]. In addition to preventing H_2_O_2_-induced oxidative stress in osteoblasts [169], chrysoeriol protected Raw264.7 macrophages from lipopolysaccharide-induced inflammation by blocking activator protein 1, which is crucial in the transcriptional activation of inducible nitric oxide synthase [170]. A hydroalcoholic extract of *Tecoma stans*, containing 96% chrysoeriol presented an enhanced activity to inhibit pancreatic lipase [171]. Relevant to the heart, chrysoeriol can lower arterial blood pressure in rats under anesthesia [97]; and it can protect against doxorubicin-induced cardiotoxicity by inhibiting apoptosis in H9c2 cells [172]. However, no published study is available on the effect of chrysoeriol on a diabetic heart at present.

#### Rooibos flavonols

Quercetin and its glycosides, quercetin-3-*O*-robinobioside, hyperoside, isoquercitrin, and rutin are classified by a distinct 3-hydroxyflavone backbone and are the major flavonols present in rooibos (Table 2). Generally, based on a specific population assessed, the average intake of flavonols may range between 20 to 35 mg/day [173, 174]. Although additional studies are required to validate their bioavailability, flavonol aglycones have been shown to be highly absorbed in the gut [175]. The type of sugar moiety and stability of aglycone largely affect the absorption of each compound as shown for quercetin glycosides from onions being better absorbed than pure aglycones [173, 176]. Another study has demonstrated that isoquercitrin and hyperoside are highly absorbable in rats [177]. Regular consumption of flavonols has been found to be protective against ischemic heart disease in some individuals [178]. Quercetin attenuated paracetamol-induced liver damage and impairment of kidney function such as intracytoplasmic vacuolization and brush border loss in rats [179]. Quercetin has a high affinity to inhibit AGEs such as methylglyoxal and glyoxal in a bovine serum albumin system [180].

Quercetin and rutin further exhibit a broad range of pharmacological activities within the myocardium (Table 1). These compounds presented atherosclerosis lowering properties by reducing hepatic fatty acid synthesis in mice [181]. They enhanced glucose uptake in muscle cells subjected to oxidative stress [182] and prevented against dyslipidemia associated complications such as inflammation and lipid toxicity by enhancing antioxidant capacity in rats [183]. Interestingly, in the heart, quercetin and rutin have been shown to directly alleviate DCM by improving myocardial ultrastructure in diabetic animals through aldose reductase, oxidative stress inhibitory activity and modulation of cardiac calcium homeostasis [184–187]. The use of quercetin at a dose of 10 mg/kg body weight for 28 days protected against autoimmune myocarditis by suppressing oxidative stress in rats [188]. Quercetin is thought to exert cardiac protection through quenching lipid peroxidation, as it is a known scavenger of peroxyl radicals [189–192]. In a double-blind randomized clinical trial on women (*n* = 72), quercetin supplementation (500 mg capsule daily) for 10 weeks significantly reduced systolic blood pressure but had no effect on other cardiovascular parameters and inflammatory biomarkers [193]. Likewise, rutin protected against myocardial damage in a diabetic state by decreasing postprandial hyperglycemia and slowing down formation of AGEs in various experimental models [194–197]. In combination with aspalathin, rutin reduced blood glucose concentrations of streptozotocin-induced diabetic rats over a 6 h monitoring period [95]. It further improved glucose homeostasis in streptozotocin-induced diabetic rats suppressing gluconeogenesis [197]. Regulation of glucose metabolism and increasing intracellular antioxidant capacity have been proposed to be the main cardioprotective effects of both quercetin and rutin [198, 199].

Like quercetin and chrysoeriol, hyperoside is often present in very low quantities in a cup of fermented rooibos tea [167]. Plants and extracts rich in hyperoside have been established to display antidiabetic properties [200–202]. Hyperoside prevented against diabetic nephropathy by inhibiting apoptosis and albuminuria in glomerular podocytes isolated from diabetic rats and mice [203, 204]. Other biological activities of hyperoside include reducing accelerated production of AGEs in ECV304 cells via the JNK pathway [205]; suppression of inflammation through reducing nuclear factor-κB activation in mouse peritoneal macrophages [206]; and inhibition of α-glucosidase and apoptosis in liver cells [207]. In the heart, hyperoside protected hyperglycemia-induced inflammation in vitro and in vivo [208]; hydrogen peroxide-induced oxidative damage [209, 210]; and it protected against ischemic-reperfusion injury in isolated rat hearts [211]. The protective mechanism of hyperoside against diabetes and heart associated complications has been mainly through suppressing cell apoptosis, improving mitochondrial function and regulating Nrf2 and extracellular signal-regulated protein kinase signaling [212, 213].

#### PPAG

PPAG is a phenylpropenoic glucoside (Table 2) that acts as a precursor in the flavonoid biosynthesis pathway and has been shown by various studies to be present in rooibos [18, 81, 108]. The occurrence of PPAG in rooibos was described for the first time about two decades ago [108] and its bioavailability profile is yet to be established. Phenylpyruvic acids apparently play a key role in the biosynthesis of a number of secondary metabolites, including PPAG [214]. The biological activity of a compound with similar structure to PPAG such as 3-phenylpyruvate has long been reported to display antidiabetic properties [215, 216]. Exposure of cardiomyocytes isolated from diabetic rats to a low concentration of fermented rooibos that contains a relatively high level of PPAG (0.71 g/100 g extract) prevents oxidative stress and apoptosis [21]. Recent findings indicated that this compound attenuates insulin resistance and protects beta cells from obese and streptozotocin-induced mice against endoplasmic reticulum stress-induced apoptosis [217–219]. Data available on the cardioprotective properties of PPAG are limited to a study in H9c2 cardiomyocytes, showing that PPAG abolishes high glucose-induced altered myocardial substrate metabolism and apoptosis by increasing the Bcl2/Bax ratio and reducing caspase 3/7 activity [21]. This study further showed that PPAG displayed reduced capacity to protect H9c2 cells against oxidative stress. This result was anticipated since PPAG is not expected to be an active antioxidant as it lacks the phenolic structural features that are required for free radical scavenging ability [220]. Interestingly, PPAG used in combination with a known antidiabetic agent such as metformin demonstrated better protection of cardiomyocytes exposed to high glucose-induced oxidative stress than when used as a monotherapy [221]. Correspondingly, Patel et al. [222] recently showed that PPAG has no inhibitory effect on cytochrome P450 enzymes, CYP2C8, CYP2C9, and CYP3A4, which are important in the metabolism of hypoglycemic drugs, such as thiazolidinediones and sulfonylureas. Supporting the potential use of nutraceutical agents such as PPAG, especially in combination with a current antidiabetic agent to attenuate oxidative stress-induced damage and protect diabetic individuals at risk of myocardial infarction needs further investigation.

## Conclusions

Blood glucose lowering therapies such as metformin and insulin have played a major role in prolonging lives of diabetic patients. However, tight control of blood glucose remains a challenge in such patients. By contrast, ameliorative therapies for oxidative stress, including polyphenols as an adjunct to current blood lowering drugs, show promise in protecting diabetic hearts in experimental models. In recent years, rooibos has gained popularity due to its potential use as a dietary supplement that is rich in polyphenols. The presence of constituents such as aspalathin, nothofagin and PPAG that are unique to rooibos or rarely occur in other plants make it attractive for scientific investigation. The compounds present in rooibos continue to present robust biological properties that are associated with ameliorative effects on inflammation and apoptosis, leading to improved cardiac function in different animal models. In addition, current evidence has suggested that the combinational use of some of these compounds with known antidiabetic agents such as metformin may enhance their biological efficacy. However, this review clearly highlights the evidence gap pertaining to the molecular mechanisms associated with the cardioprotective effect of rooibos and its polyphenols. Once these molecular mechanisms are established, in addition to verification of such findings in clinical studies, it could make a significant step in accelerating development of an evidenced-based rooibos nutraceutical. It is therefore imperative that we further investigate the mechanism(s) by which rooibos flavonoids and PPAG modulate diabetes-induced cardiovascular related complications thereby identifying new therapeutic candidates.

## Abbreviations

ACE: Angiotensin-converting enzyme
AGEs: Advanced glycation end products
AMPK: 5′-adenosine monophosphate-activated protein kinase
ARE: Antioxidant response element
CaMKII: Calmodulin kinase II
CAT: Catalase
CVD: Cardiovascular disease
DCM: Diabetic cardiomyopathy
DM: Diabetes mellitus
ERK1/2: Extracellular signal-regulated protein kinases 1 and 2
FFAs: Free fatty acids
GSH: Glutathione
H_2_O_2_: Hydrogen peroxide
HF: Heart failure
HUVECs: Human umbilical vein endothelial cells
IL-6: Interleukin-6
JNK- c: Jun N-terminal kinases
Keap1: Kelch-like ECH-associated protein 1
LDL: Low density lipoprotein
MAPK: Mitogen-activated protein kinases
MPT: Mitochondrial permeability transition
NF-kB: Nuclear factor kappa B
Nox: NADPH oxidase
Nrf2: Nuclear factor (erythroid-derived 2)-like 2
PPAG: Phenylpyruvic acid-2-*O*-β-D-glucoside
ROS: Reactive oxygen species
SOD: Superoxide dismutase

## References

[1] International Diabetes Federation (IDF). IDF Diabetes atlas 7th edition. idf.org [Internet]. Accessed at: http://www.diabetesatlas.org/. Accessed 12 December 2016.

[2] World Health Organization (WHO). Global status report on noncommunicable diseases 2014. World Health 176. 2014; doi:ISBN 9789241564854.

[3] Ginsberg BJ, Mazze R (1994). Clinical consequences of the diabetes control and complications trial. N J Med.

[4] Boudina S, Abel ED (2010). Diabetic cardiomyopathy, causes and effects. Rev Endocr Metab Disord.

[5] Kukidome D, Nishikawa T, Sonoda K, Imoto K, Fujisawa K, Yano M, Motoshima H, Taguchi T, Matsumura T, Araki E (2006). Activation of AMP-activated protein kinase reduces hyperglycemia-induced mitochondrial reactive oxygen species production and promotes mitochondrial biogenesis in human umbilical vein endothelial cells. Diabetes.

[6] Casaclang-Verzosa G, Gersh BJ, Tsang TS (2008). Structural and functional remodeling of the left atrium: Clinical and therapeutic implications for atrial fibrillation. J Am Coll Cardiol.

[7] Rubler S, Dlugash J, Yuceoglu YZ, Kumral T, Branwood AW, Grishman A (1972). New type of cardiomyopathy associated with diabetic glomerulosclerosis. Am J Cardiol.

[8] Kengne AP, Dzudie A, Sobngwi E (2008). Heart failure in sub-Saharan Africa: A literature review with emphasis on individuals with diabetes. Vasc Health Risk Manag.

[9] Mbanya JCN, Motala AA, Sobngwi E, Assah FK, Enoru ST (2010). Diabetes in sub-Saharan Africa. Lancet.

[10] Jonassen AK, Sack MN, Mjøs OD, Yellon DM (2001). Myocardial protection by insulin at reperfusion requires early administration and is mediated via Akt and p70s6 kinase cell-survival signaling. Circ Res.

[11] Eurich DT, Majumdar SR, McAlister FA, Tsuyuki RT, Johnson JA (2005). Improved clinical outcomes associated with metformin in patients with diabetes and heart failure. Diabetes Care.

[12] Yin M, Van der Horst ICC, van Melle JP, Qian C, van Gilst WH, Silljé HHW, De Boer RA (2011). Metformin improves cardiac function in a nondiabetic rat model of post-MI heart failure. Am J Physiol Heart Circ Physiol.

[13] Boudina S, Abel ED (2007). Diabetic cardiomyopathy revisited. Circulation.

[14] Maritim AC, Sanders RA, Watkins JB (2003). Diabetes, oxidative stress, and antioxidants: A review. J Biochem Mol Toxicol.

[15] Ford ES (2006). Intake and circulating concentrations of antioxidants in metabolic syndrome. Curr Atheroscler Rep.

[16] Szkudelski T, Szkudelska K (2011). Anti-diabetic effects of resveratrol. Ann N Y Acad Sci.

[17] Sellamuthu PS, Muniappan BP, Perumal SM, Kandasamy M (2009). Antihyperglycemic effect of mangiferin in streptozotocin induced diabetic rats. J Heal Sci.

[18] Muller CJ, Malherbe CJ, Chellan N, Yagasaki K, Miura Y, Joubert E. Potential of rooibos, its major *C-*glucosyl flavonoids and *Z*-2-(β-D-glucopyranoloxy)-3-phenylpropenoic acid in prevention of metabolic syndrome. Crit Rev Food Sci Nutr. 2016; doi:10.1080/10408398.2016.1157568. 10.1080/10408398.2016.1157568 27305453

[19] Tan Y, Ichikawa T, Li J, Si Q, Yang H, Chen X, Goldblatt CS, Meyer CJ, Li X, Cai L, Cui T (2011). Diabetic downregulation of Nrf2 activity via ERK contributes to oxidative stress-induced insulin resistance in cardiac cells *in vitro* and *in vivo*. Diabetes.

[20] Tarquini R, Lazzeri C, Pala L, Rotella CM, Gensini GF (2011). The diabetic cardiomyopathy. Acta Diabetol.

[21] Dludla PV, Muller CJF, Louw J, Joubert E, Salie R, Opoku AR, Johnson R (2014). The cardioprotective effect of an aqueous extract of fermented rooibos (*Aspalathus linearis*) on cultured cardiomyocytes derived from diabetic rats. Phytomedicine.

[22] Uemura S, Matsushita H, Li W, Glassford AJ, Asagami T, Lee KH, Harrison DG, Tsao PS (2001). Diabetes mellitus enhances vascular matrix metalloproteinase activity: role of oxidative stress. Circ Res.

[23] Rajamani U, Essop MF (2010). Hyperglycemia-mediated activation of the hexosamine biosynthetic pathway results in myocardial apoptosis. Am J Physiol Cell Physiol.

[24] Giacco F, Brownlee M (2010). Oxidative stress and diabetic complications. Circ Res.

[25] Cai L, Li W, Wang G, Guo L, Jiang Y, Kang YJ (2002). Hyperglycemia-induced apoptosis in mouse myocardium: Mitochondrial cytochrome c-mediated caspase-3 activation pathway. Diabetes.

[26] Sharma P, Jha AB, Dubey RS, Pessarakli M (2012). Reactive oxygen species, oxidative damage, and antioxidative defense mechanism in plants under stressful conditions. J Bot.

[27] Ansley DM, Wang B (2013). Oxidative stress and myocardial injury in the diabetic heart. J Pathol.

[28] Kuroda J, Ago T, Matsushima S, Zhai P, Schneider MD, Sadoshima J (2010). NADPH oxidase 4 (Nox4) is a major source of oxidative stress in the failing heart. Proc Natl Acad Sci U S A.

[29] Huynh K, Bernardo BC, McMullen JR, Ritchie RH (2014). Diabetic cardiomyopathy: mechanisms and new treatment strategies targeting antioxidant signaling pathways. Pharmacol Ther.

[30] Marín-García J, Akhmedov AT, Moe GW (2013). Mitochondria in heart failure: The emerging role of mitochondrial dynamics. Heart Fail Rev.

[31] Sack MN (2009). Type 2 diabetes, mitochondrial biology and the heart. J Mol Cell Cardiol.

[32] Montaigne D, Marechal X, Lefebvre P, Modine T, Fayad G, Dehondt H, Hurt C, Coisne A, Koussa M, Remy-Jouet I, Zerimech F, Boulanger E, Lacroix D, Staels B, Neviere R (2013). Mitochondrial dysfunction as an arrhythmogenic substrate: A translational proof-of-concept study in patients with metabolic syndrome developing post-operative atrial fibrillation. J Am Coll Cardiol.

[33] Zamzami N, Marchetti P, Castedo M, Decaudin D, Macho A, Hirsch T, Susin SA, Petit PX, Mignotte B, Kroemer G (1995). Sequential reduction of mitochondrial transmembrane potential and generation of reactive oxygen species in early programmed cell death. J Exp Med.

[34] Baines CP (2009). The molecular composition of the mitochondrial permeability transition pore. J Mol Cell Cardiol.

[35] Crompton M (1999). The mitochondrial permeability transition pore and its role in cell death. Biochem J.

[36] Lemasters JJ, Nieminen AL, Qian T, Trost LC, Elmore SP, Nishimura Y, Crowe RA, Cascio WE, Bradham CA, Brenner DA, Herman B (1998). The mitochondrial permeability transition in cell death: A common mechanism in necrosis, apoptosis and autophagy. Biochim Biophys Acta-Bioenerg..

[37] Kwong JQ, Molkentin JD (2015). Physiological and pathological roles of the mitochondrial permeability transition pore in the heart. Cell Metab.

[38] Galluzzi L, Bravo-San Pedro JM, Vitale I, Aaronson SA, Abrams JM, Adam D, Alnemri ES, Altucci L, Andrews D, Annicchiarico-Petruzzelli M, Baehrecke EH, Bazan NG, Bertrand MJ, Bianchi K, Blagosklonny MV, Blomgren K, Borner C, Bredesen DE, Brenner C, Campanella M, Candi E, Cecconi F, Chan FK, Chandel NS, Cheng EH, Chipuk JE, Cidlowski JA, Ciechanover A, Dawson TM, Dawson VL, De Laurenzi V, De Maria R, Debatin KM, Di Daniele N, Dixit VM, Dynlacht BD, El-Deiry WS, Fimia GM, Flavell RA, Fulda S, Garrido C, Gougeon ML, Green DR, Gronemeyer H, Hajnoczky G, Hardwick JM, Hengartner MO, Ichijo H, Joseph B, Jost PJ, Kaufmann T, Kepp O, Klionsky DJ, Knight RA, Kumar S, Lemasters JJ, Levine B, Linkermann A, Lipton SA, Lockshin RA, López-Otín C, Lugli E, Madeo F, Malorni W, Marine JC, Martin SJ, Martinou JC, Medema JP, Meier P, Melino S, Mizushima N, Moll U, Muñoz-Pinedo C, Nuñez G, Oberst A, Panaretakis T, Penninger JM, Peter ME, Piacentini M, Pinton P, Prehn JH, Puthalakath H, Rabinovich GA, Ravichandran KS, Rizzuto R, Rodrigues CM, Rubinsztein DC, Rudel T, Shi Y, Simon HU, Stockwell BR, Szabadkai G, Tait SW, Tang HL, Tavernarakis N, Tsujimoto Y, Berghe TV, Vandenabeele P, Villunger A, Wagner EF, Walczak H, White E, Wood WG, Yuan J, Zakeri Z, Zhivotovsky B, Melino G, Kroemer G (2014). Essential versus accessory aspects of cell death: Recommendations of the NCCD 2015. Cell Death Differ.

[39] Bedard K, Krause KH (2007). The NOX family of ROS-generating NADPH oxidases: Physiology and pathophysiology. Physiol Rev.

[40] Mapanga RF, Essop MF (2016). Damaging effects of hyperglycemia on cardiovascular function: Spotlight on glucose metabolic pathways. Am J Physiol Heart Circ Physiol.

[41] Paravicini TM, Touyz RM (2008). NADPH oxidases, reactive oxygen species, and hypertension: Clinical implications and therapeutic possibilities. Diabetes Care.

[42] Fu XJ, Peng YB, Hu YP, Shi YZ, Yao M, Zhang X (2014). NADPH oxidase 1 and its derived reactive oxygen species mediated tissue injury and repair. Oxidative Med Cell Longev.

[43] Stockklauser-Färber K, Ballhausen T, Laufer A, Rösen P (2000). Influence of diabetes on cardiac nitric oxide synthase expression and activity. Biochim Biophys Acta.

[44] Gray SP, Di Marco E, Okabe J, Szyndralewiez C, Heitz F, Montezano AC, De Haan JB, Koulis C, El-Osta A, Andrews KL, Chin-Dusting JPF, Touyz RM, Wingler K, Cooper ME, Schmidt HHHW, Jandeleit-Dahm KA (2013). NADPH Oxidase 1 plays a key role in diabetes mellitus-accelerated atherosclerosis. Circulation.

[45] Sukumar P, Viswambharan H, Imrie H, Cubbon RM, Yuldasheva N, Gage M, Galloway S, Skromna A, Kandavelu P, Santos CX, Gatenby VK, Smith J, Beech DJ, Wheatcroft SB, Channon KM, Shah AM, Kearney MT (2013). Nox2 NADPH oxidase has a critical role in insulin resistance-related endothelial cell dysfunction. Diabetes.

[46] Du J, Fan LM, Mai A, Li JM (2013). Crucial roles of Nox2-derived oxidative stress in deteriorating the function of insulin receptors and endothelium in dietary obesity of middle-aged mice. Br J Pharmacol.

[47] Boekema EJ, Braun HP (2007). Supramolecular structure of the mitochondrial oxidative phosphorylation system. J Biol Chem.

[48] Elmore S (2007). Apoptosis: A review of programmed cell death. Toxicol Pathol.

[49] Yu XY, Song YH, Geng YJ, Lin QX, Shan ZX, Lin SG, Li Y (2008). Glucose induces apoptosis of cardiomyocytes via microRNA-1 and IGF-1. Biochem Biophys Res Commun.

[50] An J, Chen Y, Huang Z (2004). Critical upstream signals of cytochrome c release induced by a novel Bcl-2 inhibitor. J Biol Chem.

[51] Zungu M, Schisler JC, Essop MF, McCudden C, Patterson C, Willis MS (2011). Regulation of AMPK by the ubiquitin proteasome system. Am J Pathol.

[52] Park SH, Gammon SR, Knippers JD, Paulsen SR, Rubink DS, Winder WW (2002). Phosphorylation-activity relationships of AMPK and acetyl-CoA carboxylase in muscle. J Appl Physiol.

[53] Sambandam N, Steinmetz M, Chu A, Altarejos JY, Dyck JRB, Lopaschuk GD (2004). Malonyl-CoA decarboxylase (MCD) is differentially regulated in subcellular compartments by 5’AMP-activated protein kinase (AMPK) studies using H9c2 cells overexpressing MCD and AMPK by adenoviral gene transfer technique. Eur J Biochem.

[54] Brownsey RW, Boone AN, Elliott JE, Kulpa JE, Lee WM (2006). Regulation of acetyl-CoA carboxylase. Biochem Soc Trans.

[55] Makaula S, Adam T, Essop MF (2006). Upstream stimulatory factor 1 transactivates the human gene promoter of the cardiac isoform of acetyl-CoA carboxylase. Arch Biochem Biophys.

[56] Randle PJ, Garland PB, Hales CN, Newsholme EA (1963). The glucose fatty-acid cycle its role in insulin sensitivity and the metabolic disturbances of diabetes mellitus. Lancet.

[57] Revollo J, Grimm A, Imai S (2007). The regulation of nicotinamide adenine dinucleotide biosynthesis by Nampt/PBEF/visfatin in mammals. Curr Opin.

[58] Rydström J (2006). Mitochondrial NADPH, transhydrogenase and disease. Biochim Biophys Acta-Bioenerg.

[59] Marnewick JL, Rautenbach F, Venter I, Neethling H, Blackhurst DM, Wolmarans P, MacHaria M (2011). Effects of rooibos (*Aspalathus linearis*) on oxidative stress and biochemical parameters in adults at risk for cardiovascular disease. J Ethnopharmacol.

[60] Bhatt MP, Lim YC, Hwang J, Na S, Kim YM, Ha KS (2013). C-peptide prevents hyperglycemia-induced endothelial apoptosis through inhibition of reactive oxygen species-mediated transglutaminase 2 activation. Diabetes.

[61] He X, Kan H, Cai L, Ma Q (2009). Nrf2 is critical in defense against high glucose-induced oxidative damage in cardiomyocytes. J Mol Cell Cardiol.

[62] Kobayashi A, Kang MI, Watai Y, Tong KI, Shibata T, Uchida K, Yamamoto M (2006). Oxidative and electrophilic stresses activate Nrf2 through inhibition of ubiquitination activity of Keap1. Mol Cell Biol.

[63] Bryan HK, Olayanju A, Goldring CE, Park BK (2013). The Nrf2 cell defence pathway: Keap1-dependent and -independent mechanisms of regulation. Biochem Pharmacol.

[64] Kode A, Rajendrasozhan S, Caito S, Yang SR, Megson IL, Rahman I (2008). Resveratrol induces glutathione synthesis by activation of Nrf2 and protects against cigarette smoke-mediated oxidative stress in human lung epithelial cells. Am J Physiol Lung Cell Mol Physiol.

[65] Scheen AJ (2013). Cardiovascular effects of dipeptidyl peptidase-4 inhibitors: from risk factors to clinical outcomes. Postgrad Med.

[66] Taylor F, Huffman MD, Macedo AF, Moore TH, Burke M, Davey Smith G, Ward K, Ebrahim S (2013). Statins for the primary prevention of cardiovascular disease. Cochrane Database Syst Rev.

[67] Khurana R, Malik IS (2010). Metformin: safety in cardiac patients. Postgrad Med J.

[68] Messaoudi SE, Rongen GA, de Boer RA, Riksen NP (2011). The cardioprotective effects of metformin. Curr Opin Lipidol.

[69] Bailey CJ (2008). Metformin: effects on micro and macrovascular complications in type 2 diabetes. Cardiovasc Drugs Ther.

[70] Papanas N, Maltezos E (2009). Oral antidiabetic agents: anti-atherosclerotic properties beyond glucose lowering?. Curr Pharm Des.

[71] Lexis CP, van der Horst IC, Lipsic E, Wieringa WG, de Boer RA, van den Heuvel AF, van der Werf HW, Schurer RA, Pundziute G, Tan ES, Nieuwland W, Willemsen HM, Dorhout B, Molmans BH, van der Horst-Schrivers AN, Wolffenbuttel BH, ter Horst GJ, van Rossum AC, Tijssen JG, Hillege HL, de Smet BJ, van der Harst P, van Veldhuisen DJ (2014). GIPS-III Investigators. Effect of metformin on left ventricular function after acute myocardial infarction in patients without diabetes: the GIPS-III randomized clinical trial. JAMA.

[72] Rosiak M, Postula M, Kaplon-Cieslicka A, Trzepla E, Czlonkowski A, Filipiak KJ, Opolski G (2013). Metformin treatment may be associated with decreased levels of NT-proBNP in patients with type 2 diabetes. Adv Med Sci.

[73] Johnson R, Dludla P, Joubert E, February F, Mazibuko S, Ghoor S, Muller C, Louw J (2016). Aspalathin, a dihydrochalcone *C*-glucoside, protects H9c2 cardiomyocytes against high glucose-induced shifts in substrate preference and apoptosis. Mol Nutr Food Res.

[74] Tan X, Hu J (2016). Combination therapy for type 2 diabetes: dapagliflozin plus metformin. Expert Opin Pharmacother.

[75] Falah RR, Talib WH, Shbailat SJ (2017). Combination of metformin and curcumin targets breast cancer in mice by angiogenesis inhibition, immune system modulation and induction of p53 independent apoptosis. Ther Adv Med Oncol.

[76] Erejuwa OO, Sulaiman SA, Wahab MSA, Sirajudeen KNS, Salleh MS, Gurtu S (2010). Antioxidant protective effect of glibenclamide and metformin in combination with honey in pancreas of streptozotocin-induced diabetic rats. Int J Mol Sci.

[77] Xie Z, He C, Zou MH (2011). AMP-activated protein kinase modulates cardiac autophagy in diabetic cardiomyopathy. Autophagy.

[78] Paiva MA, Rutter-Locher Z, Gonçalves LM, Providência LA, Davidson SM, Yellon DM, Mocanu MM (2011). Enhancing AMPK activation during ischemia protects the diabetic heart against reperfusion injury. Am J Physiol Heart Circ Physiol.

[79] Ríos JL, Francini F, Schinella GR (2015). Natural products for the treatment of type 2 diabetes mellitus. Planta Med.

[80] Shukla SK, Gupta S, Ojha SK, Sharma SB (2010). Cardiovascular friendly natural products: a promising approach in the management of CVD. Nat Prod Res.

[81] Joubert E, De Beer D (2011). Rooibos (*Aspalathus linearis*) beyond the farm gate: From herbal tea to potential phytopharmaceutical. S Afr J Bot.

[82] Villaño D, Pecoraria M, Testa MF, Raguzzini A, Stalmach A, Crozier A, Tubili C, Serafini M (2010). Unfermented and fermented rooibos teas (*Aspalathus linearis*) increase plasma total antioxidant capacity in healthy humans. Food Chem.

[83] Oh J, Jo H, Cho AR, Kim SJ, Han J (2013). Antioxidant and antimicrobial activities of various leafy herbal teas. Food Control.

[84] Von Gadow A, Joubert E, Hansmann CF (1997). Comparison of the antioxidant activity of rooibos tea (*Aspalathus linearis*) with green, oolong and black tea. Food Chem.

[85] Joubert E, Winterton P, Britz TJ, Ferreira D (2004). Superoxide anion and α, α-diphenyl-β-picrylhydrazyl radical scavenging capacity of rooibos (*Aspalathus linearis*) aqueous extracts, crude phenolic fractions, tannin and flavonoids. Food Res Int.

[86] Waisundara VY, Hoon LY (2015). Free radical scavenging ability of *Aspalathus linearis* in two *in vitro* models of diabetes and cancer. J Tradit Complement Med.

[87] Ulicná O, Vancová O, Bozek P, Cársky J, Sebeková K, Boor P, Nakano M, Greksák M (2006). Rooibos tea (*Aspalathus linearis*) partially prevents oxidative stress in streptozotocin-induced diabetic rats. Physiol Res.

[88] Hong IS, Lee HY, Kim HP (2014). Anti-oxidative effects of Rooibos tea (*Aspalathus linearis*) on immobilization-induced oxidative stress in rat brain. PLoS One.

[89] Ajuwon OR, Marnewick JL, Davids LM. Rooibos (*Aspalathus linearis*) and its major flavonoids – potential against oxidative stress-induced conditions. InTech 2015;http://doi.org/10.5772/61614.

[90] Sanderson M, Mazibuko SE, Joubert E, De Beer D, Johnson R, Pheiffer C, Louw J, Muller CJF (2014). Effects of fermented rooibos (*Aspalathus linearis*) on adipocyte differentiation. Phytomedicine.

[91] Mazibuko SE. In vitro and in vivo effect of *Aspalathus linearis* and its major polyphenols on carbohydrate and lipid metabolism in insulin resistant models. Doctoral dissertation, University of Zululand, 2014. Accessed 12 December 2016.

[92] Baba H, Ohtsuka Y, Haruna H, Lee T, Nagata S, Maeda M, Yamashiro Y, Shimizu T (2009). Studies of anti-inflammatory effects of rooibos tea in rats. Pediatr Int.

[93] Beltrán-Debón R, Rull A, Rodríguez-Sanabria F, Iswaldi I, Herranz-López M, Aragonès G, Camps J, Alonso-Villaverde C, Menéndez JA, Micol V, Segura-Carretero A, Joven J (2011). Continuous administration of polyphenols from aqueous rooibos (*Aspalathus linearis*) extract ameliorates dietary-induced metabolic disturbances in hyperlipidemic mice. Phytomedicine.

[94] Smith C, Swart AC (2016). Rooibos (*Aspalathus linearis*) facilitates an anti-inflammatory state, modulating IL-6 and IL-10 while not inhibiting the acute glucocorticoid response to a mild novel stressor *in vivo*. J Funct Foods.

[95] Muller CJF, Joubert E, De Beer D, Sanderson M, Malherbe CJ, Fey SJ, Louw J (2012). Acute assessment of an aspalathin-enriched green rooibos (*Aspalathus linearis*) extract with hypoglycemic potential. Phytomedicine.

[96] Kamakura R, Son MJ, De Beer D, Joubert E, Miura Y, Yagasaki K (2015). Antidiabetic effect of green rooibos (*Aspalathus linearis*) extract in cultured cells and type 2 diabetic model KK-A(y) mice. Cytotechnology.

[97] Khan AU, Gilani AH (2006). Selective bronchodilatory effect of rooibos tea (*Aspalathus linearis*) and its flavonoid, chrysoeriol. Eur J Nutr.

[98] Persson IA, Persson K, Hägg S, Andersson RG (2010). Effects of green tea, black tea and rooibos tea on angiotensin-converting enzyme and nitric oxide in healthy volunteers. Public Health Nutr.

[99] Persson IA, Josefsson M, Persson K, Andersson RG (2006). Tea flavanols inhibit angiotensin-converting enzyme activity and increase nitric oxide production in human endothelial cells. J Pharm Pharmacol.

[100] Persson IA (2012). The pharmacological mechanism of angiotensin-converting enzyme inhibition by green tea, rooibos and enalaprilat-a study on enzyme kinetics. Phytother Res.

[101] Pantsi WG, Marnewick JL, Esterhuyse AJ, Rautenbach F, van Rooyen J (2011). Rooibos (*Aspalathus linearis*) offers cardiac protection against ischaemia/reperfusion in the isolated perfused rat heart. Phytomedicine.

[102] Tangney CC, Rasmussen HE (2013). Polyphenols, inflammation, and cardiovascular disease. Curr Atheroscler Rep.

[103] Bors W, Heller W, Michel C, Saran M (1990). Flavonoids as antioxidants: determination of radical-scavenging efficiencies. Methods Enzymol.

[104] Xiao J, Kai G (2012). A review of dietary polyphenol-plasma protein interactions: characterization, influence on the bioactivity, and structure-affinity relationship. Crit Rev Food Sci Nutr.

[105] Xiao J, Kai G, Yamamoto K, Chen X (2013). Advance in dietary polyphenols as α-glucosidases inhibitors: a review on structure-activity relationship aspect. Crit Rev Food Sci Nutr.

[106] Xiao J. Dietary flavonoid aglycones and their glycosides: Which show better biological significance? Crit Rev Food Sci Nutr. 2015; doi: 10.1080/10408398.2015.1032400. 10.1080/10408398.2015.1032400 26176651

[107] Beelders T, Sigge GO, Joubert E, de Beer D, de Villiers A (2012). Kinetic optimisation of the reversed phase liquid chromatographic separation of rooibos tea (*Aspalathus linearis*) phenolics on conventional high performance liquid chromatographic instrumentation. J Chromatogr A.

[108] Marais C, Steenkamp JA, Ferreira D. Occurrence of phenylpyruvic acid in woody plants: Biosynthetic significance and synthesis of an enolic glucoside derivative. J Chem Soc Perkin Trans. 1. 1996;(24):2915-2918.

[109] Stalmach A, Mullen W, Pecorari M, Serafini M, Crozier A (2009). Bioavailability of *C-*linked dihydrochalcone and flavanone glucosides in humans following ingestion of unfermented and fermented rooibos teas. J Agric Food Chem.

[110] Joubert E, De Beer D. Antioxidants of rooibos beverages: role of plant composition and processing. In: Preedy V. Processing and impact on antioxidants in beverages, 1^st^ edition. Academic Press; 2014. p. 131–143.

[111] Courts FL, Williamson G (2015). The occurrence, fate and biological activities of *C*-glycosyl flavonoids in the human diet. Crit Rev Food Sci Nutr.

[112] Breiter T, Laue C, Kressel G, Gröll S, Engelhardt UH, Hahn A (2011). Bioavailability and antioxidant potential of rooibos flavonoids in humans following the consumption of different rooibos formulations. Food Chem.

[113] Cuervo A, Hevia A, López P, Suárez A, Sánchez B, Margolles A, González S (2015). Association of polyphenols from oranges and apples with specific intestinal microorganisms in systemic lupus erythematosus patients. Nutrients..

[114] Ozdal T, Sela DA, Xiao J, Boyacioglu D, Chen F, Capanoglu E (2016). The reciprocal interactions between polyphenols and gut microbiota and effects on bioaccessibility. Nutrients..

[115] Koutsos A, Tuohy KM, Lovegrove JA (2015). Apples and cardiovascular health--is the gut microbiota a core consideration?. Nutrients.

[116] Bowles S, Joubert E, De Beer D, Louw J, Brunschwig C, Njoroge M, Lawrence N, Wiesner L, Chibale K, Muller C (2017). Intestinal transport characteristics and metabolism of C-glucosyl dihydrochalcone, aspalathin. Molecules.

[117] Snijman PW, Joubert E, Ferreira D, Li XC, Ding Y, Green IR, Gelderblom WC (2009). Antioxidant activity of the dihydrochalcones aspalathin and nothofagin and their corresponding flavones in relation to other rooibos (*Aspalathus linearis*) flavonoids, epigallocatechin gallate, and trolox. J Agric Food Chem.

[118] Ku SK, Kwak S, Kim Y, Bae JS (2015). Aspalathin and nothofagin from rooibos (*Aspalathus linearis*) inhibits high glucose-induced inflammation *in vitro* and *in vivo*. Inflammation.

[119] Ku SK, Lee W, Kang M, Bae JS (2015). Antithrombotic activities of aspalathin and nothofagin via inhibiting platelet aggregation and FIIa/FXa. Arch Pharm Res.

[120] Najafian M, Najafian B, Najafian Z (2016). The effect of aspalathin on levels of sugar and lipids in streptozotocin-induced diabetic and normal rats. Zahedan J Res Med Sci.

[121] Kawano A, Nakamura H, Hata S, Minakawa M, Miura Y, Yagasaki K (2009). Hypoglycemic effect of aspalathin, a rooibos tea component from *Aspalathus linearis*, in type 2 diabetic model *db/db* mice. Phytomedicine.

[122] Son MJ, Minakawa M, Miura Y, Yagasaki K (2013). Aspalathin improves hyperglycemia and glucose intolerance in obese diabetic ob/ob mice. Eur J Nutr.

[123] Mazibuko SE, Joubert E, Johnson R, Louw J, Opoku AR, Muller CJ (2015). Aspalathin improves glucose and lipid metabolism in 3T3-L1 adipocytes exposed to palmitate. Mol Nutr Food Res.

[124] Johnson R, Dludla PV, Muller CJ, Huisamen B, Essop MF, Louw J (2017). The transcription profile unveils the cardioprotective effect of aspalathin against lipid toxicity in an *in vitro* H9c2 model. Molecules.

[125] Smit SE (2016). An investigation into the effects of aspalathin on myocardial glucose transport using cardiomyocytes, and terminally differentiated H9c2 cells.

[126] Dludla PV, Muller CJF, Joubert E, Louw J, Essop MF, Gabuza KB, Ghoor S, Huisamen B, Johnson R (2017). Aspalathin protects the heart against hyperglycemia-induced oxidative damage by up-regulating Nrf2 expression. Molecules.

[127] Joubert E, Beelders T, de Beer D, Malherbe CJ, de Villiers AJ, Sigge GO (2012). Variation in phenolic content and antioxidant activity of fermented rooibos herbal tea infusions: Role of production season and quality grade. J Agric Food Chem.

[128] Shi J, Zhu L, Li Y, Zheng H, Yu J, Lu L, Liu Z (2016). *In vitro* study of UGTs metabolism and permeability of orientin and isoorientin, two active flavonoid *C-*glycosides. Drug Metab Lett.

[129] Liu L, Guo L, Zhao C, Wu X, Wang R, Liu C (2015). Characterization of the intestinal absorption of seven flavonoids from the flowers of *Trollius chinensis* using the Caco-2 cell monolayer model. PLoS One.

[130] Li D, Wang Q, Yuan ZF, Zhang L, Xu L, Cui Y, Duan K (2008). Pharmacokinetics and tissue distribution study of orientin in rat by liquid chromatography. J Pharm Biomed Anal.

[131] Zhou P, Li LP, Luo SQ, Jiang HD, Zeng S (2008). Intestinal absorption of luteolin from peanut hull extract is more efficient than that from individual pure luteolin. J Agric Food Chem.

[132] Likhitwitayawuid K, Klongsiriwet C, Jongbunprasert V, Sritularak B, Wongseripipatana S (2006). Flavones with free radical scavenging activity from *Goniothalamus tenuifolius*. Arch Pharm Res.

[133] Kim J, Lee I, Seo J, Jung M, Kim Y, Yim N, Bae K (2010). Vitexin, orientin and other flavonoids from *Spirodela polyrhiza* inhibit adipogenesis in 3T3-L1 cells. Phytother Res.

[134] Ku SK, Kwak S, Bae JS (2014). Orientin inhibits high glucose-induced vascular inflammation *in vitro* and *in vivo*. Inflammation.

[135] Orrego R, Leiva E, Cheel J (2009). Inhibitory effect of three *C-*glycosylflavonoids from *Cymbopogon citratus* (Lemongrass) on human low density lipoprotein oxidation. Molecules.

[136] Liu LY, Ma QQ, Li JY, Xu J, Yuan L, Li JP, Huang XL (2013). The therapeutic effect of orientin on myocardial infarction rats. Lishizhen Medicine and Materia Medica Research.

[137] Liu L, Wu Y, Huang X (2016). Orientin protects myocardial cells against hypoxia-reoxygenation injury through induction of autophagy. Eur J Pharmacol.

[138] Lu N, Sun Y, Zheng X (2011). Orientin-induced cardioprotection against reperfusion is associated with attenuation of mitochondrial permeability transition. Planta Med.

[139] Lam KY, Ling AP, Koh RY, Wong YP, Say YH (2016). A review on medicinal properties of orientin. Adv Pharmacol Sci.

[140] Fu XC, Wang MW, Li SP, Wang HL (2006). Anti-apoptotic effect and the mechanism of orientin on ischaemic/reperfused myocardium. J Asian Nat Prod Res.

[141] Yuan L, Han X, Li W, Ren D, Yang X (2016). Isoorientin prevents hyperlipidemia and liver injury by regulating lipid metabolism, antioxidant capability, and inflammatory cytokine release in high-fructose-fed mice. J Agric Food Chem.

[142] Lim JH, Park HS, Choi JK, Lee IS, Choi HJ (2007). Isoorientin induces Nrf2 pathway-driven antioxidant response through phosphatidylinositol 3-kinase signaling. Arch Pharm Res.

[143] Choo CY, Sulong NY, Man F, Wong TW (2012). Vitexin and isovitexin from the leaves of *Ficus deltoidea* with *in-vivo* α-glucosidase inhibition. J Ethnopharmacol.

[144] Yao Y, Cheng X, Wang L, Wang S, Ren G (2011). A determination of potential α-glucosidase inhibitors from Azuki Beans (*Vigna angularis*). Int J Mol Sci.

[145] Peng X, Zheng Z, Cheng KW, Shan F, Ren GX, Chen F, Wang M (2008). Inhibitory effect of mung bean extract and its constituents vitexin and isovitexin on the formation of advanced glycation end products. Food Chem.

[146] Dong L, Fan Y, Shao X, Chen Z (2011). Vitexin protects against myocardial ischemia/reperfusion injury in Langendorff-perfused rat hearts by attenuating inflammatory response and apoptosis. Food Chem Toxicol.

[147] Wang Y, Zhen Y, Wu X, Jiang Q, Li X, Chen Z, Zhang G, Dong L (2015). Vitexin protects brain against ischemia/reperfusion injury via modulating mitogen-activated protein kinase and apoptosis signaling in mice. Phytomedicine.

[148] Dong LY, Li S, Zhen YL, Wang YN, Shao X, Luo ZG (2013). Cardioprotection of vitexin on myocardial ischemia/reperfusion injury in rat via regulating inflammatory cytokines and MAPK pathway. Am J Chin Med..

[149] Wang XS, Hu XC, Chen GL, Yuan X, Yang RN, Liang S, Ren J, Sun JC, Kong GQ, Gao SG, Feng XS (2015). Effects of vitexin on the pharmacokinetics and mRNA expression of CYP isozymes in rats. Phytother Res.

[150] Shao X, Dong LY, Li XL, Guo Y, Jiang Q, Fang M (2010). Effects of vitexin on hemodynamics and myocardial oxygen consumption in anesthetized dogs. Anhui Med and Pharm J.

[151] Lu CC, Xu YQ, Wu JC, Hang PZ, Wang Y, Wang C, Wu JW, Qi JC, Zhang Y, Du ZM (2013). Vitexin protects against cardiac hypertrophy via inhibiting calcineurin and CaMKII signaling pathways. Naunyn Schmiedeberg's Arch Pharmacol.

[152] Dong LY, Chen ZW, Guo Y, Cheng XP, Shao X (2008). Mechanisms of vitexin preconditioning effects on cultured neonatal rat cardiomyocytes with anoxia and reoxygenation. Am J Chin Med..

[153] Kwon EY, Jung UJ, Park T, Yun JW, Choi MS (2015). Luteolin attenuates hepatic steatosis and insulin resistance through the interplay between the liver and adipose tissue in mice with diet-induced obesity. Diabetes.

[154] Liu Y, Tian X, Gou L, Sun L, Ling X, Yin X (2013). Luteolin attenuates diabetes-associated cognitive decline in rats. Brain Res Bull.

[155] Wang GG, Lu XH, Li W, Zhao X, Zhang C (2011). Protective effects of luteolin on diabetic nephropathy in STZ-induced diabetic rats. Evid Based Complement Alternat Med.

[156] Lamy S, Moldovan PL, Ben Saad A, Annabi B (2015). Biphasic effects of luteolin on interleukin-1β-induced cyclooxygenase-2 expression in glioblastoma cells. Biochim Biophys Acta.

[157] Bagli E, Stefaniotou M, Morbidelli L, Ziche M, Psillas K, Murphy C, Fotsis T (2004). Luteolin inhibits vascular endothelial growth factor-induced angiogenesis; inhibition of endothelial cell survival and proliferation by targeting phosphatidylinositol 3′-kinase activity. Cancer Res.

[158] Weng Z, Patel AB, Vasiadi M, Therianou A, Theoharides TC (2014). Luteolin inhibits human keratinocyte activation and decreases NF-κB induction that is increased in psoriatic skin. PLoS One.

[159] Abdalla S, Zarga MA, Sabri S (1994). Effects of the flavone luteolin, isolated from *Colchicum richii*, on guinea-pig isolated smooth muscle and heart and on blood pressure and blood flow. Phytother Res.

[160] Su J, Xu HT, Yu JJ, Gao JL, Lei J, Yin QS, Li B, Pang MX, Su MX, Mi WJ, Chen SH, Lv GY (2015). Luteolin ameliorates hypertensive vascular remodeling through inhibiting the proliferation and migration of vascular smooth muscle cells. Evid Based Complement Alternat Med.

[161] El-Bassossy HM, Abo-Warda SM, Fahmy A (2014). Chrysin and luteolin alleviate vascular complications associated with insulin resistance mainly through PPAR-γ activation. Am J Chin Med..

[162] Bian C, Xu T, Zhu H, Pan D, Liu Y, Luo Y, Wu P, Li D (2015). Luteolin inhibits Ischemia/reperfusion-induced myocardial injury in rats via downregulation of microRNA-208b-3p. PLoS One.

[163] Wang G, Li W, Lu X, Bao P, Zhao X (2012). Luteolin ameliorates cardiac failure in type I diabetic cardiomyopathy. J Diabetes Complicat.

[164] Sun GB, Sun X, Wang M, Ye JX, Si JY, Xu HB, Meng XB, Qin M, Sun J, Wang HW, Sun XB (2012). Oxidative stress suppression by luteolin-induced heme oxygenase-1 expression. Toxicol Appl Pharmacol.

[165] Wu X, Xu T, Li D, Zhu S, Chen Q, Hu W, Pan D, Zhu H, Sun H (2013). ERK/PP1a/PLB/SERCA2a and JNK pathways are involved in luteolin-mediated protection of rat hearts and cardiomyocytes following ischemia/reperfusion. PLoS One.

[166] Odontuya G, Hoult JR, Houghton PJ (2005). Structure-activity relationship for antiinflammatory effect of luteolin and its derived glycosides. Phytother Res.

[167] Toyoda M, Tanaka K, Hoshino K, Akiyama H, Tanimura A, Saito Y (1997). Profiles of potentially antiallergic flavonoids in 27 kinds of health tea and green tea infusions. J Agric Food Chem.

[168] Mishra B, Priyadarsini KI, Kumar MS, Unnikrishnan MK, Mohan H (2003). Effect of *O*-glycosilation on the antioxidant activity and free radical reactions of a plant flavonoid, chrysoeriol. Bioorg Med Chem.

[169] Kim YH, Lee YS, Choi EM (2010). Chrysoeriol isolated from Eurya cilliata leaves protects MC3T3-E1 cells against hydrogen peroxide-induced inhibition of osteoblastic differentiation. J Appl Toxicol.

[170] Choi DY, Lee JY, Kim MR, Woo ER, Kim YG, Kang KW (2005). Chrysoeriol potently inhibits the induction of nitric oxide synthase by blocking AP-1 activation. J Biomed Sci.

[171] Ramirez G, Zamilpa A, Zavala M, Perez J, Morales D, Tortoriello J (2016). Chrysoeriol and other polyphenols from *Tecoma stans* with lipase inhibitory activity. J Ethnopharmacol.

[172] Liu Z, Song XD, Xin Y, Wang XJ, Yu H, Bai YY, Liu JH, Zhang CN, Hui RT (2009). Protective effect of chrysoeriol against doxorubicin-induced cardiotoxicity *in vitro*. Chin Med J.

[173] Hollman PC, Katan MB (1999). Dietary flavonoids: intake, health effects and bioavailability. Food Chem Toxicol.

[174] Vogiatzoglou A, Mulligan AA, Lentjes MA, Luben RN, Spencer JP, Schroeter H, Khaw KT, Kuhnle GG (2015). Flavonoid intake in European adults (18 to 64 years). PLoS One.

[175] Scalbert A, Morand C, Manach C, Rémésy C (2002). Absorption and metabolism of polyphenols in the gut and impact on health. Biomed Pharmacother.

[176] Hollman PC, De Vries JH, Van Leeuwen SD, Mengelers MJ, Katan MB (1995). Absorption of dietary quercetin glycosides and quercetin in healthy ileostomy volunteers. Am J Clin Nutr.

[177] Chang Q, Zuo Z, Chow MS, Ho WK (2005). Difference in absorption of the two structurally similar flavonoid glycosides, hyperoside and isoquercitrin, in rats. Eur J Pharm Biopharm.

[178] Hertog MG, Feskens EJ, Hollman PC, Katan MB, Kromhout D (1993). Dietary antioxidant flavonoids and risk of coronary heart disease: the Zutphen Elderly Study. Lancet.

[179] Yousef MI, Omar SA, El-Guendi MI, Abdelmegid LA (2010). Potential protective effects of quercetin and curcumin on paracetamol-induced histological changes, oxidative stress, impaired liver and kidney functions and haematotoxicity in rat. Food Chem Toxicol.

[180] Li X, Zheng T, Sang S, Lv L (2014). Quercetin inhibits advanced glycation end product formation by trapping methylglyoxal and glyoxal. J Agric Food Chem.

[181] Odbayar TO, Badamhand D, Kimura T, Takashi Y, Tsushida T, Ide T (2006). Comparative studies of some phenolic compounds (quercetin, rutin, and ferulic acid) affecting hepatic fatty acid synthesis in mice. J Agric Food Chem.

[182] Dhanya R, Arun KB, Syama HP, Nisha P, Sundaresan A, Santhosh Kumar TR, Jayamurthy P (2014). Rutin and quercetin enhance glucose uptake in L6 myotubes under oxidative stress induced by tertiary butyl hydrogen peroxide. Food Chem.

[183] Hu QH, Zhang X, Pan Y, Li YC, Kong LD (2012). Allopurinol, quercetin and rutin ameliorate renal NLRP3 inflammasome activation and lipid accumulation in fructose-fed rats. Biochem Pharmacol.

[184] Krishna KM, Annapurna A, Gopal GS, Chalam CR, Madan K, Kumar VK, Prakash GJ (2005). Partial reversal by rutin and quercetin of impaired cardiac function in streptozotocin-induced diabetic rats. Can J Physiol Pharmacol.

[185] Wang YB, Ge ZM, Kang WQ, Lian ZX, Yao J, Zhou CY (2015). Rutin alleviates diabetic cardiomyopathy in a rat model of type 2 diabetes. Exp Ther Med..

[186] Guimaraes JFC, Muzio BP, Rosa CM, Nascimento AF, Sugizaki MM, Fernandes AAH, Cicogna AC, Padovani CR, Okoshi MP, Okoshi K (2015). Rutin administration attenuates myocardial dysfunction in diabetic rats. Cardiovasc Diabetol.

[187] Mahmoud MF, Hassan NA, El Bassossy HM, Fahmy A (2013). Quercetin protects against diabetes-induced exaggerated vasoconstriction in rats: effect on low grade inflammation. PLoS One.

[188] Milenković M, Arsenović-Ranin N, Stojić-Vukanić Z, Bufan B, Vučićević D, Jančić I (2010). Quercetin ameliorates experimental autoimmune myocarditis in rats. J Pharm Pharm Sci.

[189] Cai Q, Rahn RO, Zhang R (1997). Dietary flavonoids, quercetin, luteolin and genistein, reduce oxidative DNA damage and lipid peroxidation and quench free radicals. Cancer Lett.

[190] Psotová J, Chlopcíková S, Grambal F, Simánek V, Ulrichová J (2002). Influence of silymarin and its flavonolignans on doxorubicin-iron induced lipid peroxidation in rat heart microsomes and mitochondria in comparison with quercetin. Phytother Res.

[191] Meyer AS, Heinonen M, Frankel EN (1998). Antioxidant interactions of catechin, cyanidin, caffeic acid, quercetin, and ellagic acid on human LDL oxidation. Food Chem.

[192] Kamada C, da Silva EL, Ohnishi-Kameyama M, Moon JH, Terao J (2005). Attenuation of lipid peroxidation and hyperlipidemia by quercetin glucoside in the aorta of high cholesterol-fed rabbit. Free Radic Res.

[193] Zahedi M, Ghiasvand R, Feizi A, Asgari G, Darvish L (2013). Does quercetin improve cardiovascular risk factors and inflammatory biomarkers in women with type 2 diabetes: a double-blind randomized controlled clinical trial. Int J Prev Med.

[194] Fernandes AA, Novelli EL, Okoshi K, Okoshi MP, Di Muzio BP, Guimarães JF, Fernandes JA (2010). Influence of rutin treatment on biochemical alterations in experimental diabetes. Biomed Pharmacother.

[195] Pashikanti S, de Alba DR, Boissonneault GA, Cervantes-Laurean D (2010). Rutin metabolites: novel inhibitors of nonoxidative advanced glycation end products. Free Radic Biol Med.

[196] Cervantes-Laurean D, Schramm DD, Jacobson EL, Halaweish I, Bruckner GG, Boissonneault GA (2006). Inhibition of advanced glycation end product formation on collagen by rutin and its metabolites. J Nutr Biochem.

[197] Prince P, Kamalakkannan N (2006). Rutin improves glucose homeostasis in streptozotocin diabetic tissues by altering glycolytic and gluconeogenic enzymes. J Biochem Mol Toxicol.

[198] Ahn J, Lee H, Kim S, Park J, Ha T (2008). The anti-obesity effect of quercetin is mediated by the AMPK and MAPK signaling pathways. Biochem Biophys Res Commun.

[199] Umarani V, Muvvala S, Ramesh A, Lakshmi BV, Sravanthi N (2015). Rutin potentially attenuates fluoride-induced oxidative stress-mediated cardiotoxicity, blood toxicity and dyslipidemia in rats. Toxicol Mech Methods.

[200] Liang T, Yue W, Li Q (2010). Comparison of the phenolic content and antioxidant activities of *Apocynum venetum L.* (Luo-Bu-Ma) and two of its alternative species. Int J Mol Sci.

[201] Dezsi S, Bădărău AS, Bischin C, Vodnar DC, Silaghi-Dumitrescu R, Gheldiu AM, Mocan A, Vlase L (2015). Antimicrobial and antioxidant activities and phenolic profile of *Eucalyptus globulus Labill*. and *Corymbia ficifolia* (F. Muell.) K.D. Hill & L.A.S. Johnson leaves. Molecules.

[202] Wang SS, Wang DM, Pu WJ, Li DW (2013). Phytochemical profiles, antioxidant and antimicrobial activities of three *Potentilla* species. BMC Complement Altern Med.

[203] Zhou L, An XF, Teng SC, Liu JS, Shang WB, Zhang AH, Yuan YG, Yu JY (2012). Pretreatment with the total flavone glycosides of *Flos Abelmoschus manihot* and hyperoside prevents glomerular podocyte apoptosis in streptozotocin-induced diabetic nephropathy. J Med Food.

[204] Zhang J, Fu H, Xu Y, Niu Y, An X (2016). Hyperoside reduces albuminuria in diabetic nephropathy at the early stage through ameliorating renal damage and podocyte injury. J Nat Med.

[205] Zhang Z, Sethiel MS, Shen W, Liao S, Zou Y (2013). Hyperoside downregulates the receptor for advanced glycation end products (RAGE) and promotes proliferation in ECV304 cells via the c-Jun N-terminal kinases (JNK) pathway following stimulation by advanced glycation end-products *in vitro*. Int J Mol Sci.

[206] Kim SJ, Um JY, Lee JY (2011). Anti-inflammatory activity of hyperoside through the suppression of nuclear factor-κB activation in mouse peritoneal macrophages. Am J Chin Med.

[207] Liu X, Zhu L, Tan J, Zhou X, Xiao L, Yang X, Wang B (2014). Glucosidase inhibitory activity and antioxidant activity of flavonoid compound and triterpenoid compound from *Agrimonia Pilosa Ledeb*. BMC Complement Altern Med.

[208] Ku SK, Kwak S, Kwon OJ, Bae JS (2014). Hyperoside inhibits high-glucose-induced vascular inflammation *in vitro* and *in vivo*. Inflammation.

[209] Li ZL, Liu JC, Hu J, Li XQ, Wang SW, Yi DH, Zhao MG (2012). Protective effects of hyperoside against human umbilical vein endothelial cell damage induced by hydrogen peroxide. J Ethnopharmacol.

[210] Piao MJ, Kang KA, Zhang R, Ko DO, Wang ZH, You HJ, Kim HS, Kim JS, Kang SS, Hyun JW (2008). Hyperoside prevents oxidative damage induced by hydrogen peroxide in lung fibroblast cells via an antioxidant effect. Biochim Biophys Acta.

[211] Han J, Xuan JL, Hu HR, Chen ZW (2015). Protective effect against myocardial ischemia reperfusion injuries induced by hyperoside preconditioning and its relationship with PI3K/Akt signaling pathway in rats. Zhongguo Zhong Yao Za Zhi.

[212] Hou JY, Liu Y, Liu L, Li XM (2016). Protective effect of hyperoside on cardiac ischemia reperfusion injury through inhibition of ER stress and activation of Nrf2 signaling. Asian Pac J Trop Med.

[213] Li ZL, Hu J, Li YL, Xue F, Zhang L, Xie JQ, Liu ZH, Li H, Yi DH, Liu JC, Wang SW (2013). The effect of hyperoside on the functional recovery of the ischemic/reperfused isolated rat heart: potential involvement of the extracellular signal-regulated kinase 1/2 signaling pathway. Free Radic Biol Med.

[214] Joubert E, De Beer D, Malherbe CJ, Muller N, Bonnet SL, Van Der Westhuizen JH, Ferreira D (2013). Occurrence and sensory perception of Z-2-(β-D-glucopyranosyloxy)-3- phenylpropenoic acid in rooibos (*Aspalathus linearis*). Food Chem.

[215] Malaisse WJ, Sener A, Welsh M, Malaisse-Lagae F, Hellerstrom C, Christophe J (1983). Mechanism of 3-phenylpyruvate-induced insulin release. Metabolic aspects Biochem J.

[216] Ben-Abraham R, Gazit V, Vofsi O, Ben-Shlomo I, Reznick AZ, Katz Y (2003). Beta-phenylpyruvate and glucose uptake in isolated mouse soleus muscle and cultured C2C12 muscle cells. J Cell Biochem.

[217] Muller CJF, Joubert E, Pheiffer C, Ghoor S, Sanderson M, Chellan N, Fey SJ, Louw J (2013). Z-2-(β-d-glucopyranosyloxy)-3-phenylpropenoic acid, an α-hydroxy acid from rooibos (*Aspalathus linearis*) with hypoglycemic activity. Mol Nutr Food Res.

[218] Mathijs I, da Cunha DA, Himpe E, Ladriere L, Chellan N, Roux CR, Joubert E, Muller C, Cnop M, Louw J, Bouwens L (2014). Phenylpropenoic acid glucoside augments pancreatic beta cell mass in high-fat diet-fed mice and protects beta cells from ER stress-induced apoptosis. Mol Nutr Food Res.

[219] Himpe E, Cunha DA, Song I, Bugliani M, Marchetti P, Cnop M, Bouwens L (2016). Phenylpropenoic acid glucoside from rooibos protects pancreatic beta cells against cell death induced by acute injury. PLoS One.

[220] Orzel J, Daszykowski M, Kazura M, De Beer D, Joubert E, Schulze AE, Beelders T, De Villiers A, Malherbe CJ, Walczak B (2014). Modeling of the total antioxidant capacity of rooibos (*Aspalathus linearis*) tea infusions from chromatographic fingerprints and identification of potential antioxidant markers. J Chromatogr A.

[221] Dludla PV, Muller CJ, Joubert E, Louw J, Gabuza KB, Huisamen B, Essop MF, Johnson R (2016). Phenylpyruvic acid-2-*O*-β-D-glucoside attenuates high glucose-induced apoptosis in H9c2 cardiomyocytes. Planta Med.

[222] Patel O, Muller C, Joubert E, Louw J, Rosenkranz B, Awortwe C (2016). Inhibitory Interactions of *Aspalathus linearis* (rooibos) extracts and compounds, aspalathin and Z-2-(β-D-glucopyranosyloxy)-3-phenylpropenoic acid, on cytochromes metabolizing hypoglycemic and hypolipidemic drugs. Molecules.

[223] Lee W, Ku SK, Bae JS (2014). Vascular barrier protective effects of orientin and isoorientin in LPS-induced inflammation *in vitro* and *in vivo*. Vasc Pharmacol.

[224] Che X, Wang X, Zhang J, Peng C, Zhen Y, Shao X, Zhang G, Dong L (2016). Vitexin exerts cardioprotective effect on chronic myocardial ischemia/reperfusion injury in rats via inhibiting myocardial apoptosis and lipid peroxidation. Am J Transl Res.

[225] Occhiuto F, Circosta C, De Pasquale A, Briguglio F (1990). Comparative haemodynamic effects of the flavonoids rhoifolin and vitexin in the dog. Phytother Res.

[226] Sun Z, Yan B, Yu WY, Yao X, Ma X, Sheng G, Ma Q (2016). Vitexin attenuates acute doxorubicin cardiotoxicity in rats via the suppression of oxidative stress, inflammation and apoptosis and the activation of FOXO3a. Exp Ther Med.

[227] Jiang H, Xia Q, Wang X, Song J, Bruce IC (2005). Luteolin induces vasorelaxion in rat thoracic aorta via calcium and potassium channels. Pharmazie.

[228] Liao PH, Hung LM, Chen YH, Kuan YH, Zhang FB, Lin RH, Shih HC, Tsai SK, Huang SS (2011). Cardioprotective effects of luteolin during ischemia-reperfusion injury in rats. Circ J.

[229] Challa SR, Akula A, Metla S, Gopal PN (2011). Partial role of nitric oxide in infarct size limiting effect of quercetin and rutin against ischemia-reperfusion injury in normal and diabetic rats. Indian J Exp Biol.

[230] Galindo P, Rodriguez-Gómez I, González-Manzano S, Dueñas M, Jiménez R, Menéndez C, Vargas F, Tamargo J, Santos-Buelga C, Pérez-Vizcaíno F, Duarte J (2012). Glucuronidated quercetin lowers blood pressure in spontaneously hypertensive rats via deconjugation. PLoS One.

[231] Guo XD, Zhang DY, Gao XJ, Parry J, Liu K, Liu BL, Wang M (2013). Quercetin and quercetin-3-O-glucuronide are equally effective in ameliorating endothelial insulin resistance through inhibition of reactive oxygen species-associated inflammation. Mol Nutr Food Res.

[232] Annapurna A, Reddy CS, Akondi RB, Rao SR (2009). Cardioprotective actions of two bioflavonoids, quercetin and rutin, in experimental myocardial infarction in both normal and streptozotocin-induced type I diabetic rats. J Pharm Pharmacol.

[233] Li M, Jiang Y, Jing W, Sun B, Miao C, Ren L (2013). Quercetin provides greater cardioprotective effect than its glycoside derivative rutin on isoproterenol-induced cardiac fibrosis in the rat. Can J Physiol Pharmacol.

[234] Panchal SK, Poudyal H, Arumugam TV, Brown L (2011). Rutin attenuates metabolic changes, nonalcoholic steatohepatitis, and cardiovascular remodeling in high-carbohydrate, high-fat diet-fed rats. J Nutr.

[235] Saklani R, Gupta SK, Mohanty IR, Kumar B, Srivastava S, Mathur R (2016). Cardioprotective effects of rutin via alteration in TNF-α, CRP, and BNP levels coupled with antioxidant effect in STZ-induced diabetic rats. Mol Cell Biochem.

